# Genetic Analysis Reveals Climate Exposure and Conservation Opportunities for a Vulnerable Life History in Steelhead ( *Oncorhynchus mykiss* )

**DOI:** 10.1111/eva.70293

**Published:** 2026-07-10

**Authors:** Michelle Y. Pepping, Tasha Q. Thompson, Sean M. O'Rourke, Jonathan L. Hart, Michael R. Miller, Matthew R. Sloat

**Affiliations:** ^1^ Department of Animal Science University of California, Davis Davis California USA; ^2^ Wild Salmon Center Portland Oregon USA

**Keywords:** climate change, conservation genetics, intraspecific diversity, migration, mykiss, steelhead

## Abstract

The loss of intraspecific diversity is a hidden crisis that threatens to disrupt ecological processes and ecosystem services, diminishing economic, subsistence, and cultural benefits to human communities. Conserving this diversity requires a deeper understanding of its evolutionary underpinnings and the ecological conditions necessary for its persistence amid rapid environmental change. Here, we integrate evolutionary principles and empirical evidence from recently developed genetic assays to examine the maintenance of run timing diversity within steelhead ( *Oncorhynchus mykiss* ), a widely distributed anadromous salmonid with distinct summer and winter adult migration timings. Using a GREB1L‐based genetic assay, we analyzed nearly 2000 juvenile samples from the North Umpqua River, Oregon, USA to map watershed‐scale distributions of run timing genotypes alongside climate‐driven stream warming patterns. We found that summer‐run genotypes dominate only in habitats located above seasonally passable barriers, such as natural waterfalls, that restrict or prevent passage by adult winter‐run steelhead. This pattern supports the hypothesis that exclusive access to habitat provides a critical fitness advantage that compensates for the costs associated with summer run timing. Temperature modeling further revealed that habitats currently exclusive to summer‐run steelhead are already thermally stressful and are projected to become increasingly so under end‐of‐century climate scenarios, reducing the quality of critical summer‐run steelhead habitat. Because the persistence of summer‐run steelhead under climate change depends on access to exclusive habitat of sufficient quality, we propose strategies to establish or maintain habitat exclusivity in more climate‐resilient areas within the basin while minimizing impacts on winter‐run steelhead and other anadromous species. More broadly, our study demonstrates how understanding the genetic basis of run timing and the fitness trade‐offs associated with different life histories can inform the conservation of intraspecific diversity in migratory fish.

## 1 Introduction

Intraspecific diversity encompasses genetic and phenotypic variation within and among populations (Hilborn et al. 2003; Schindler et al. 2010; Des Roches et al. 2018). This diversity arises and is maintained through mechanisms such as environmental and habitat heterogeneity, where alternative phenotypes are favored across different temporal or spatial contexts. Intraspecific diversity enhances short‐term population resilience through the portfolio effect while simultaneously providing the evolutionary foundation for long‐term species persistence and adaptation (Hilborn et al. 2003; Greene et al. 2009; Braun et al. 2016). However, threats to intraspecific diversity abound, including local extirpations, population declines, and anthropogenic selection pressure (Moore et al. 2010; Carlson and Satterthwaite 2011; Mimura et al. 2017; Thompson et al. 2019). These losses occur at rates many times greater than species‐level extinctions and can precipitate ultimate extinction (Des Roches et al. 2021). Loss of intraspecific diversity also disrupts ecological processes and ecosystem services, and can diminish benefits to human societies (Des Roches et al. 2018). Developing effective conservation measures to preserve intraspecific diversity requires understanding both the natural selective forces that establish this variation and contemporary stressors that threaten it.

Intraspecific diversity is exceptionally high in *Oncorhynchus mykiss* , a salmonid widely distributed across the North Pacific Rim that exhibits both a freshwater resident form (rainbow trout) and an anadromous form (steelhead) (Quinn 2007). Steelhead reproduce in freshwater streams where juveniles typically rear for 1–2 years before migrating to the ocean to feed for one or more years, after which they return to their natal streams for reproduction. A crucial component of steelhead life history diversity is the seasonal timing of their breeding migration, also known as run timing (Withler 1966; Quinn et al. 2016; Waples et al. 2022). In much of their North American range, distinct steelhead runs are commonly referred to by the season when they return to freshwater: “winter‐run” steelhead enter rivers from late fall through winter in an advanced stage of sexual maturation and spawn shortly after reaching their natal spawning grounds; “summer‐run” steelhead enter rivers from spring through early summer in a relatively sexually immature state and complete maturation while holding in freshwater for up to ten months before spawning. Run timing diversity allows the species to hedge against temporal environmental variation and diversifies spatial habitat use by facilitating access to stream habitat that can only be reached during seasons with suitable flow levels (Quinn et al. 2016).

Despite the species‐level benefits of run timing variation, the extended period of adult freshwater holding of summer‐run steelhead makes them particularly vulnerable to human activities, including direct harvest effects and indirect impacts from land use changes and climate‐driven alterations to summer stream temperatures and baseflows (Quinn et al. 2016). Consequently, anthropogenic impacts have disproportionately affected summer‐run populations across much of their range (Busby et al. 1996; Moyle et al. 2008; Waples et al. 2022). Recent declines have prompted emergency fishing regulations and complete fishery closures for summer‐run steelhead in several North American populations, including in watersheds where winter‐run populations remain stable or are increasing (Barker 2021; ODFW 2022, 2023). This pattern underscores an urgent need to develop conservation strategies specifically targeting the summer‐run life history.

Based on both theory and empirical evidence, a critical component of summer‐run steelhead persistence is thought to be access to exclusive habitat that is inaccessible to the winter‐run (Quinn et al. 2016; Kannry et al. 2020; Waples et al. 2022). The summer‐run life history incurs substantial costs that must be balanced by its benefits. Early freshwater entry requires summer‐run steelhead to forgo months of ocean foraging opportunities and invest twice as much energy in lipid storage for their extended freshwater residence, resulting in smaller body size and reduced fecundity compared to winter‐run fish (Quinn et al. 2011; Lamperth et al. 2017; Waples et al. 2022). In addition, warm summer stream temperatures challenge summer‐run steelhead survival and maturation, as steelhead metabolic rates are heavily influenced by water temperature (Keefer et al. 2009, 2018; Dressler et al. 2025). Thus, summer‐run steelhead are at a disadvantage when required to directly compete on spawning grounds with larger, more fecund winter‐run steelhead. However, by entering rivers during summer and early fall, summer‐run steelhead experience low flow conditions that enable passage at cascades and falls that can become impassable during high winter flows (Quinn et al. 2016). This limits direct competition between summer‐run and winter‐run steelhead for spawning and rearing habitat and provides the benefit of exclusive (or near‐exclusive) habitat access that offsets the costs of the summer‐run life history.

Understanding the function of flow‐dependent barriers and protecting, restoring, or expanding upstream habitat therefore presents a critical need for summer‐run steelhead conservation efforts (Kannry et al. 2020). However, determining whether a putative flow‐dependent barrier is truly effective in creating exclusive summer‐run habitat (i.e., in limiting winter‐run passage) has, until recently, been challenged by the lack of outwardly distinguishable traits between summer‐run and winter‐run steelhead during spawning and juvenile rearing. Although the general distribution of summer‐run adults has been documented in many rivers through methods such as seasonal dive surveys, the distribution of juveniles and the degree of overlap with winter‐run steelhead have remained unclear. Recent genomic discoveries have improved the ability to map summer‐run habitat use by revealing that steelhead run timing is controlled by a major‐effect locus (*GREB1L*), where individuals homozygous for the early‐run variant exhibit summer‐run timing, those homozygous for the late‐run variant exhibit winter‐run timing, and heterozygotes display intermediate timing (Hess et al. 2016; Prince et al. 2017; Waples et al. 2022). This discovery has enabled development of genetic assays to efficiently determine the run‐type (i.e., summer vs. winter vs. heterozygous) genotype of any individual, including juveniles. These molecular tools can now address previously unanswered questions about summer‐run steelhead ecology and inform targeted conservation strategies (Kannry et al. 2020; Fraik et al. 2021; Waples et al. 2022).

**FIGURE 1:**
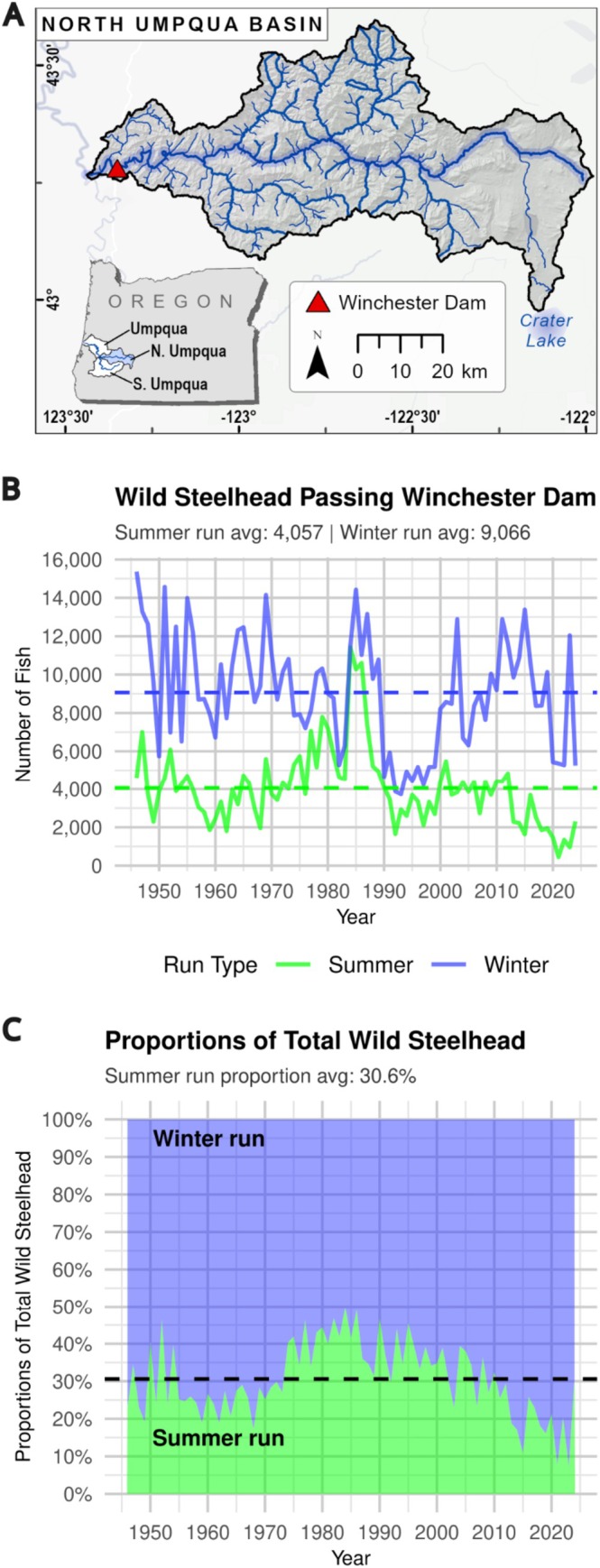
The North Umpqua River Basin and wild steelhead runs. (A) Map of the North Umpqua Basin. (B) The number of adult wild steelhead migrating past Winchester Dam each year from 1946 to 2024. Fish are counted as summer run (green) from May 1 to November 30, and are counted as winter run (blue) from December 1 to April 30. Dashed lines indicate the average across all years for each run. Winter‐run fish are considered to belong to the year during which the winter migration season began (e.g., all winter‐run fish in the December 1, 2018 to April 30, 2019 season are counted as 2018 winter‐run fish). Counting methods changed from partial day monitoring to 24‐h video monitoring in 1992, and the correction developed in McGie (1994) has been applied to counts before that date. Counts from 2015 to 2024 are estimates based on subsampled video monitoring calibrated with previous years. (C) Proportions of total wild steelhead passing Winchester Dam assigned to each run. The correction applied in part B is also applied here.

Summer‐run steelhead in the North Umpqua River, Oregon, USA (Figure 1) represent a population where conservation strategies informed by genetic tools and understanding of the ecological factors maintaining the summer‐run life history carry the potential to reverse recent declines and improve population resilience. The North Umpqua River hosts one of the largest remaining summer‐run populations alongside an even larger winter‐run population (ODFW 2022). It supports a recreational fishery that attracts anglers from around the world and contributes significantly to the local rural economy (Blumm and Kloos 1986). A fish ladder on Winchester Dam, located just above the North Umpqua's confluence with the South Umpqua, has enabled abundance estimates of both run types since 1946 (Figure 1). From 1946 to 2024, estimates at the dam have averaged 4057 wild summer‐run (2.5–97.5 percentiles: 1327–10289) and 9066 wild winter‐run steelhead (2.5–97.5 percentiles: 4221–14,432) annually (McGie 1994; ODFW 2025b, 2025c). However, summer‐run populations have experienced sharp declines in recent years, both in absolute numbers and relative to winter‐run abundance (Figure 1). The four lowest adult summer‐run estimates on record have all occurred since 2020, including the only 2 years with fewer than 1000 fish (2021: 450; 2023: 960). These declines prompted emergency fishing closures in 2021 and 2023—the first such closures since Oregon began regulating North Umpqua fishing (ODFW 2022, 2023).

Here, we have applied genomic tools and other approaches to address three objectives: (1) map the distribution of summer‐run and winter‐run steelhead juveniles in the North Umpqua and evaluate the relationship with putative flow‐dependent barriers; (2) explore the feasibility of specific management actions to expand exclusive summer‐run steelhead habitat; (3) identify current and future thermal exposure of summer‐run steelhead habitat. First, we genetically analyzed almost 2000 juvenile samples from sites distributed across the North Umpqua basin to evaluate the role of putative flow‐dependent barriers in summer‐run ecology and precisely define critical summer‐run habitat. Next, we explored the suitability and potential for recently reopened habitat above an upper basin dam to be managed to benefit summer‐run steelhead. Finally, we evaluated stream temperatures in both current and potential summer‐run habitat and their projected changes under climate scenarios to assess future habitat viability for summer‐run steelhead.

## 2 Methods

### 2.1 Study System

The North Umpqua River (Figure 1) is a fifth order stream in southern Oregon, USA that flows west from the Cascade Range to the confluence with the South Umpqua River to form the Umpqua River, which drains to the Pacific Ocean. Most of the 3500 km^2^ basin is owned by the United States Forest Service, the Bureau of Land Management, and private land owners (Palmer 2014).

**FIGURE 2:**
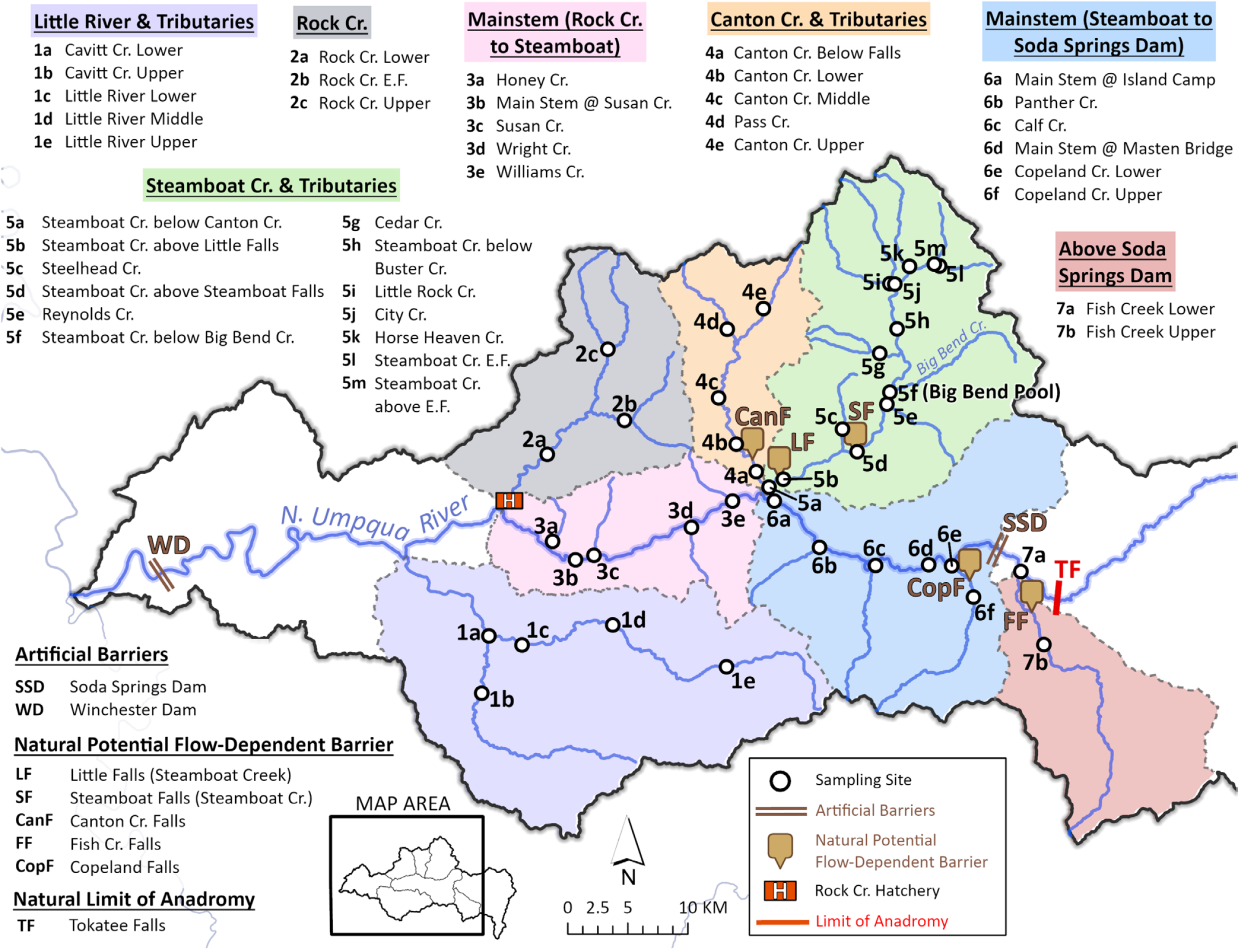
Map of the North Umpqua Basin showing juvenile *O. mykiss* sampling locations and natural and artificial barriers. Winchester Dam (WD) is passable via fish ladder. Soda Spring Dam (SSD) blocked fish passage after its construction in 1952 until a fish ladder was added in 2012. Natural potential flow‐dependent barriers are falls, cascades, or velocity barriers that may impede or block steelhead access at high flows (see Supporting Information).

The North Umpqua features multiple natural falls and cascades hypothesized to function as flow‐dependent barriers to steelhead migration (Box 1). Two prominent examples, Little Falls and Steamboat Falls, are located on Steamboat Creek, a southwest‐flowing tributary joining the North Umpqua at river kilometer (rkm) 85 (Figure 2) that supports by far the largest number of oversummering adult summer‐run steelhead of any tributary in the basin (Loomis et al. 2003). Black Gorge, a deep canyon located between Little and Steamboat Falls, also contains a waterfall, as well as narrow constrictions that may potentially be hydraulic barriers (sensu Wright et al. 2024) where upstream fish migration is prevented by high water velocities at high flows (Box 1). Canton Creek (a Steamboat Creek tributary), Copeland Creek, and Fish Creek also feature falls or steep cascades that may impede winter‐run access during high flows (Box 1; Figure 2). However, the function of these barriers is less well documented than those on Steamboat Creek. By sampling juvenile steelhead above and below these potential barriers across multiple years, we aimed to clarify their roles in creating exclusive summer‐run habitat.

BOX 1 **Description of natural and anthropogenic seasonal barriers to anadromous fish migration in the North Umpqua basin, Oregon, USA.**

**Natural Potential Flow‐Dependent Barriers**

**Steamboat Falls (Steamboat Creek)**

Steamboat Falls, a ~ 7.5 m two‐step waterfall located 10 rkm above Steamboat Creek's confluence with the North Umpqua, is believed to have historically been a natural flow‐dependent barrier that restricted winter‐run steelhead passage. However, the falls were modified with the demolition of a portion of the falls and the addition of a fish ladder in 1959 Loomis et al. (2003), which was further modified in 2012. The modifications to Steamboat Falls were intended to enable fish passage across a wider range of flows. However, the enclosed design of the ladder resulted in substantial debris accumulations during winter flows that had to be manually removed once flows had subsided. This may have resulted in the continued exclusion of winter‐run steelhead above the falls. Modifications to the ladder in 2012 were intended to eliminate winter debris accumulation and facilitate winter passage, but it is unclear if those goals were met.

**Little Falls (Steamboat Creek)**

Little Falls is a 2.5 m bedrock step located 2.3 rkm above Steamboat Creek's confluence with the North Umpqua Loomis et al. (2003). It is believed to be impassable at certain flows, but the degree to which it impedes winter‐run steelhead passage is unclear.

**Black Gorge (Steamboat Creek)**

Black Gorge is a deep canyon that begins approximately 1.5 km above Little Falls and ends approximately 2.5 km below Steamboat Falls. It is difficult to access and not as well described as the other barriers. However, the gorge contains narrow constrictions and an approximately 2.5 m waterfall that is considered a mandatory portage for kayakers. In addition, a telemetry study showed migrating adult steelhead spent considerable time in Black Gorge (Loomis et al. 2003), although whether this was due to the difficulty of passage or the presence of holding habitat was not explored.

**Canton Creek Falls (Canton Creek)**

A series of falls in lower Canton Creek may be impassable to adult steelhead at certain flows. The most substantial is Canton Creek Falls, a 1.8 m bedrock step located about 2.25 rkm above Canton Creek's confluence with Steamboat Creek Loomis et al. (2003). Canton Creek Falls is a barrier to Coho salmon, which migrate in the late fall and winter, but the extent to which it may impede winter‐run steelhead is less clear.

**Copeland Falls (Copeland Creek)**

Copeland Falls, also called Fountain of Youth Falls, is a ~ 4.5 m step located 2.8 rkm above Copeland Creek's confluence with the North Umpqua. It may act as a barrier at certain flows, but it is unclear to what extent it impedes winter‐run steelhead passage.

**Fish Creek Falls (Fish Creek)**

Fish Creek Falls is a 4.2 m waterfall formed by large boulders 5.1 rkm upstream of the Fish Creek mouth (Lewis 2013). It is located within a steep, narrow canyon with many boulder fields and cascades that may themselves be impassable at high flows. Soda Springs Dam blocked anadromous migration to Fish Creek from 1952 until 2012 when a fish ladder was constructed to bypass the dam. While steelhead redds have been observed in lower Fish Creek since construction of the ladder (ODFW 2025a), it is unknown whether any steelhead have ascended the canyon and falls. A Forest Service Diary written prior to the construction of Soda Springs dam reportedly recorded observations of steelhead in Fish Creek upstream of the falls, suggesting historical utilization of that habitat by steelhead (Coates 2012).

**Artificial Barriers**

**Winchester Dam**

Winchester dam is a 4.9 m high concrete dam on the North Umpqua mainstem located 11.3 rkm above the confluence with the South Umpqua River. While the dam went through several earlier iterations, the current structure was constructed in 1907. Originally used for hydroelectric power, the dam is now solely used to create a recreational lake for a local waterski club. A fish ladder facilitates passage, and fish counts have been recorded at the ladder since 1946.

**Soda Springs Dam**

A 23.5 m high diversion dam completed in 1952 and located at 112 rkm on the North Umpqua mainstem (ODFW 2025a). The dam blocked anadromous passage from its construction until a fish ladder was added in 2012. Currently, the fish ladder is open and passable all year. This manuscript explores whether the ladder could feasibly be managed to create exclusive summer‐run habitat (i.e., exclude winter‐run steelhead) above the dam without significant impacts on other species.

The North Umpqua and its tributaries also contain hydrological features that are critical for oversummering summer‐run steelhead. Tributary confluences in Steamboat Creek create thermal refuges for the large number of adult summer‐run steelhead that over‐summer there (Baigún 2003). Most notably, Big Bend Pool, located just below the confluence with Big Bend Creek (Figure 2), maintains cool summer water temperatures relative to elsewhere in Steamboat Creek where temperatures stressful to steelhead are frequently exceeded (e.g., > 20°C; Keefer et al. 2009; Keefer et al. 2018). It is common for several hundred or more steelhead to over‐summer in this single pool (Loomis et al. 2003; Spencer 2017). However, these refuges are spatially restricted and their capacity to retain cool temperatures with climate warming is unclear. The loss of these cold‐water refuges would fundamentally disrupt current summer‐run steelhead production. Therefore, we assessed the vulnerability of these refuges under climate change and evaluated potential alternative summer‐run steelhead habitat within the North Umpqua basin under both current and projected conditions.

**FIGURE 3:**
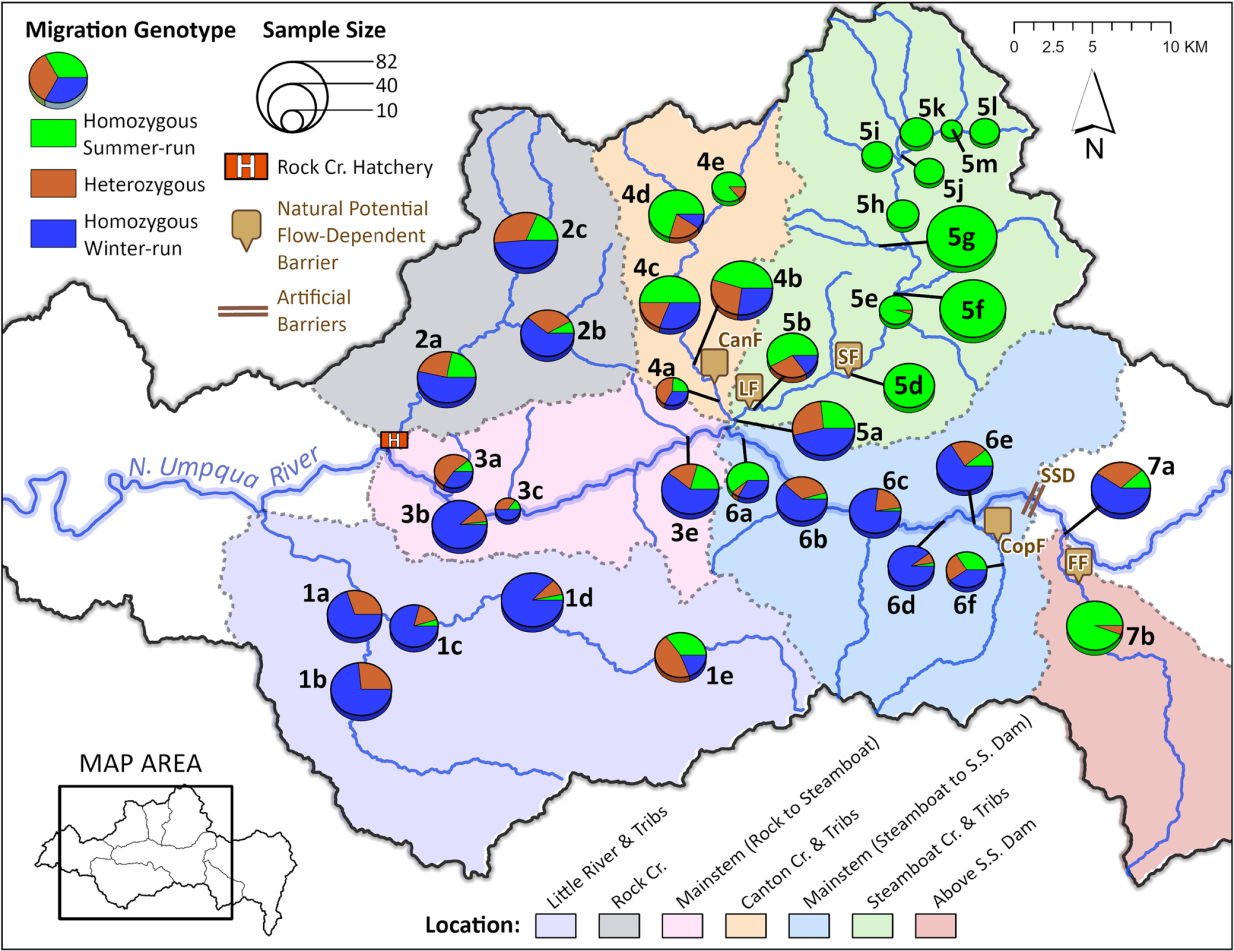
Run timing genotype proportions at juvenile *O. mykiss* sampling locations throughout the North Umpqua Basin. Pie chart position indicates sampling location. In some cases, a black line connects the pie chart to its precise sampling location. Pie chart size indicates the number of samples collected at the location across all sampling years (2017–2019). Colors indicate the proportions of each *GREB1L* genotype found at a location. Labels correspond to the location codes listed in Figure 2.

Soda Springs Dam on the upper mainstem North Umpqua (Figure 2; Figure 3) blocked anadromous fish passage from 1952 until the addition of a fish ladder in 2012 (ODFW 2025a). Since construction of the ladder, anadromous species including steelhead, spring Chinook salmon ( *Oncorhynchus tshawytscha* ), and coho salmon ( *Oncorhynchus kisutch* ) have begun recolonizing the habitat. Between 11 and 105 wild summer‐run steelhead and 139–375 winter‐run steelhead have been observed passing the dam each year (ODFW 2025a). At least 11 km of spawning habitat is known to exist above the dam, much of which is in lower Fish Creek (Figure 2). Fifty to sixty‐five kilometers of additional habitat would be available if Fish Creek Falls (Figures 2 and 3) is passable to summer‐run steelhead, but the difficulty of ascending the falls may make recolonization a relatively slow process, especially if summer‐run steelhead are not numerous in the habitat below the falls. While it is not known if steelhead currently migrate above Fish Creek Falls, *O. mykiss* are present above the falls and could persist as a non‐anadromous, stream resident population (discussed in more detail below).

The Soda Springs fish ladder could potentially be managed to expand exclusive summer‐run steelhead habitat to the area above the dam by excluding passage during the time winter‐run steelhead are ascending to spawning grounds (i.e., the ladder could be managed to be a seasonal barrier). This would be expected to increase the abundance of summer‐run steelhead above the dam, which in turn may improve the likelihood and speed of recolonization above Fish Creek Falls. However, such a management action would require careful consideration of impacts on other species and on winter‐run steelhead.

Artificial propagation may also influence steelhead dynamics in the North Umpqua basin (ODFW 2022). A hatchery on lower Rock Creek (Figure 2) produced summer‐run steelhead until its destruction in a 2020 wildfire but was operational throughout our sample collection period. Although hatchery steelhead are known to stray into the river system above Rock Creek, very few hatchery summer‐run steelhead appear to stray into Steamboat Creek, as adipose‐clipped fish are rarely observed during surveys there (ODFW 2022). The number of hatchery‐origin summer‐run steelhead observed passing above Soda Springs Dam on the upper mainstem varies greatly between years but sometimes equals or exceeds the number of wild summer‐run steelhead passing the dam (ODFW 2025a).

### 2.2 Sample Collection

We collected juvenile *O. mykiss* samples at locations throughout the North Umpqua Basin from Little River to Fish Creek (Figure 2) from 2017 to 2019 under NOAA 4 (d) permit numbers 20,885, 21,794, and 2266, and under University of California, Davis IACUC protocol number 19350. We sampled each location once per year during August and September. We used dip nets with up to 29″ × 34″ hoop width and 44″ depth made of rubber or fine nylon mesh, employing both stationary and sweeping techniques to target juvenile *O. mykiss* . Collections occurred at night when fish are less likely to be concealed by cover and water temperatures are cooler, both of which make fish easier to capture. Following capture, we visually identified individuals to species and immediately released fish that were not identified as steelhead. For fish visually identified as steelhead, we clipped a small portion of the caudal fin, measured the fish's fork length, and then released the fish. We placed fin clips on Whatman paper and stored them air‐dried in a coin envelope until laboratory processing. We sampled approximately 20 individuals at each of about 30 locations per year (Figure 2; Figure S1). Sampling locations varied slightly between years to adjust to areas inaccessible due to wildfires, to reduce off‐target species sampling, and minimize sibling collection (see below).

### 2.3 DNA Extraction, Sequencing, and Initial Data Processing

We transferred a portion of each juvenile fin clip to a well in a 96‐well plate and recorded plate and well locations for each sample. We extracted DNA using an Ampure XP bead‐based protocol as described in Ali et al. (2016) with an additional heating step at 55°C for one minute during final DNA elution. We quantified DNA using Quant‐iT PicoGreen dsDNA Reagent (Thermo Fisher Scientific) with an FLx800 Fluorescence Reader (BioTek Instruments).

We generated SbfI RAD‐capture (Rapture) libraries for all 2017 and 2018 samples using the protocol and 500 bait sequences designed for *O. mykiss* described in Ali et al. (2016). This protocol combines restriction‐site associated DNA (RAD) sequencing with capture‐based enrichment via biotinylated RNA baits targeting specific RAD loci previously identified as informative for *O. mykiss* population structure (Miller et al. 2007; Baird et al. 2008; Ali et al. 2016). The bait sequences targeted loci distributed across the genome and included two baits for a RAD locus within the *GREB1L* region associated with adult migration timing (Prince et al. 2017). However, *GREB1L* genotype calls for all samples in this study were made with a *GREB1L* Taqman‐based qPCR assay (see below) because it was applied to samples from all years and the rate of missing calls was lower than in the *GREB1L* Rapture bait data. Each sample received a unique combination of plate and well barcodes. Libraries were sequenced on an Illumina HiSeq 4000 to generate 150‐bp paired‐end reads, with 2017 and 2018 samples occupying 20% and 50% of a lane, respectively. Samples collected in 2019 did not undergo RAD‐capture sequencing and instead were genotyped only with the qPCR assay (see below).

Sequence data were demultiplexed, requiring perfect barcode and partial restriction enzyme site matches as described in Ali et al. (2016), then aligned to the rainbow trout genome assembly (Omyk_1.0, GCA_002163495.1) (Pearse et al. 2019) using BWA with the mem algorithm (Li 2013). We used Samtools to remove duplicates, retain only properly paired reads, and sort the resulting BAM files (Danecek et al. 2021).

### 2.4 Sample Quality Control

While juvenile fish were field‐identified to species via visual inspection prior to tissue sampling, it is difficult to visually distinguish juvenile *O. mykiss* from juvenile coastal cutthroat trout ( *Oncorhynchus clarkii* ), both of which are abundant in the North Umpqua basin. Additionally, Chinook salmon and coho salmon are present in most of our sampling areas. To inform 2019 sampling location selection and ensure results would not be biased by off‐target species inclusion, we performed principal component analysis (PCA) on Rapture sequencing data from 2017 and 2018 samples. We also included publicly available sequence data from the other salmonid species known to occur in the basin (Table S1): two cutthroat trout, two Chinook salmon, and two coho salmon.

We only analyzed samples with at least 1000 properly paired reads. We used the software program ANGSD (version 0.935) to perform the analysis (Korneliussen et al. 2014). We excluded reads that mapped to chromosome 5 of the *O. mykiss* genome because that chromosome is known to segregate a very large inversion that can create bias in PCA analyses (Miller et al. 2012). Only data that mapped uniquely (‐uniqueOnly 1) and with a minimum base quality score of 20 and a minimum mapping quality score of 20 were included. Quality scores around indels were recalculated with ‐baq 1 (Li 2011b). Major and minor alleles were determined from genotype likelihoods (−doMajorMinor 1) using the Samtools model (−GL 1) (Li 2011a). Loci were included if they had a SNP *p*‐value of 10^−6^ or lower (‐SNP_pval 0.000001) and minimum minor allele frequency of 0.05 (−doMaf 3, −minMaf 0.05) (Kim et al. 2011). Only SNPs with data present in at least 50% of samples (‐minInd N) were used. This resulted in 17,937 SNPS passing filtering. Single read sampling was used to construct a covariance matrix (‐doIBS 1, ‐doCounts 1, ‐doCov 1). Single read sampling effectively downsamples each individual to 1X coverage at each locus, reducing bias from coverage variability between samples. PCA on the covariance matrix was performed and visualized in R using custom scripts (R Core Team 2024).

The results of the PCA clearly identified samples that were not *O. mykiss* (see Results; Figure S2; Dataset S1). We excluded these individuals from subsequent analyses. In addition, the results informed sample site selection for 2019. Any locations with > 15% cutthroat trout in 2017 and/or 2018 were excluded from the 2019 dataset but were retained for 2017 and 2018 with the off‐target samples removed (Figure S3; Dataset S1). Cutthroat trout, Chinook salmon, and coho salmon are fixed for the steelhead summer‐run variant at the *GREB1L* SNP used in this study, so the inclusion of any off‐target samples would inflate the number of summer‐run alleles reported.

### 2.5 GREB1L Genotyping

To genotype run timing variation in the *GREB1L* region, we designed a qPCR assay based on the approach used in Thompson et al. (2019) for another salmonid species (Table S2). Briefly, biotinylated RNA baits were previously designed with affinities across the *GREB1L* region and used for capture‐based enrichment and sequencing of the region in 25 summer‐run and 24 winter‐run steelhead from 10 rivers in Northern California, Oregon, and Washington (including the North Umpqua) as described in Prince et al. (2017). Diagnostic SNPs for migration time were determined based on the strength of association in all river populations included in that study. We designed a qPCR TaqMan‐based SNP Genotyping Assay (Schleinitz et al. 2011) for one of the diagnostic SNPs (chromosome 28, position 21,528,503; Omyk_1.0, GCA_002163495.1). We applied the assay to samples from Prince et al. (2017) to validate efficacy and concordance.

Next, we applied this assay to all juvenile samples collected from the North Umpqua basin to assign a run timing genotype to each sample. To be included in downstream analyses, genotypes had to be called before the 28th qPCR cycle.

### 2.6 Spatial and Temporal Analyzes of Run Timing Genetic Variation

We evaluated spatial patterns associated with run timing genetic variation by calculating *GREB1L* genotype frequencies for each sampling location. We then graphed and plotted genotype frequencies onto a map to identify patterns related to geography and putative flow‐dependent barriers. We evaluated the interannual variability of allele frequencies at each site through pairwise comparisons among all sample years.

### 2.7 Analysis of Population Structure and Relationship With Run Timing

To characterize the population structure of *O. mykiss* in the North Umpqua basin, we performed PCA on Rapture data from 2017 and 2018 samples. We applied the same filtering criteria described for off‐target species identification, except that only samples with at least 10,000 reads that had been successfully genotyped at *GREB1L* were included and reads mapping to chromosomes 5 (which contains a large genomic region with high linkage disequilibrium known to impact population structure analysis) and chromosome 28 (which contains *GREB1L*) were excluded. A total of 12,689 SNPs passed filtering. PCA was performed on the resulting covariance matrix and visualized using custom R scripts (R Core Team 2024), with *GREB1L* genotype information overlaid to examine the relationship between population structure and run timing. The initial PCA revealed an outlier population (Upper Fish Creek; see Results), so for the sake of clearly visualizing the structure in the rest of the basin, we repeated the analysis with these samples removed and then repeated the analysis a third time with Copeland Creek samples removed. We report output from all three iterations in the Results section.

### 2.8 Feasibility of Managing Soda Spring Dam Fish Ladder to Provide Exclusive Summer‐Run Steelhead Habitat by Excluding Winter‐Run Steelhead

To explore the potential impacts on other species of seasonally closing the Soda Springs fish ladder to winter‐run steelhead passage, we analyzed public data collected by the operators of the fish ladder to evaluate the number and timing of each anadromous species passing the dam. The data included counts of all species observed passing the ladder in each month of the year between 2013 and 2024. For each species (or run type, in the case of steelhead), we calculated the proportion of the total wild counts that passed during each month, aggregated across all years. Next, we evaluated whether a time period existed for closing the ladder that could effectively exclude most winter‐run steelhead but not affect the passage of other anadromous species.

To evaluate potential population‐level impacts of closing the Soda Springs ladder to winter‐run steelhead passage, we calculated the number of wild winter‐run steelhead passing Soda Spring Dam each year as a proportion of total winter‐run steelhead counts at Winchester Dam as reported by the managing agency (ODFW 2025a, 2025c) (Figure 1).

### 2.9 Evaluation of Stream Temperatures Under Current and Future Conditions

To characterize summer stream temperatures in summer‐run steelhead habitat, we deployed a network of continuously recording, calibrated temperature data loggers (HOBO Pendant MX, accuracy ±0.5°C; Onset Computer Corp., Pocasset, Massachusetts) and retrieved water temperature data from three USGS hydrological stations. We also deployed two air temperature data loggers to examine air‐water temperature relationships as an indicator of stream temperature sensitivity to climate warming. Water and air temperature data loggers were programmed to record at 15‐min intervals. Water temperature loggers were installed in well‐mixed stream locations, and all data loggers were shielded from direct solar radiation using perforated opaque plastic sleeves. All temperature data loggers were deployed from June 19 to September 15, 2024. We summarized temperature logger and USGS stream gage data into daily maximum, minimum, and mean temperatures. We also evaluated the number of days at each location where maximum daily water temperature exceeded 20°C, a threshold for temperature‐induced physiological stress in steelhead (Keefer et al. 2009, 2018). At some temperature logger locations, longer time series of water temperature data were available from the United States Forest Service (USFS). Where this was the case, we also calculated 10‐year averages for maximum daily maximum water temperature (MDMT; i.e., the warmest annual water temperature measurement) and the average annual number of days that maximum daily water temperature exceeded 20°C.

To model stream temperature changes under a climate warming scenario, we first evaluated the relationship between stream and air temperature at each data logger location by plotting daily maximum water temperatures against the corresponding daily mean air temperature from the nearest air temperature logger site. Using R, we fit individual Ordinary Least Squares (OLS) linear models at each site and calculated Pearson's correlation coefficient to evaluate the strength and direction of the linear relationship (R Core Team 2024).

We used the estimated linear relationship between maximum water and mean air temperatures from 2024 to estimate future stream temperatures under a 3.5°C air temperature warming scenario. A 3.5°C warming projection was chosen to approximate a likely end‐of‐century scenario. This projection is between the minimum projected warming for Oregon by 2074 (2.7°C) and 2100 (4.2°C), assuming limited greenhouse gas reductions (Fleishman 2025). New values for daily maximum water temperatures were predicted using the site‐specific linear models, updating the independent variable (mean daily air temperature) with the +3.5°C increase. These predicted future temperatures were then used to estimate the increase in the number of days that summer water temperatures may exceed 20°C in a year otherwise similar to 2024. This approach assumes no change in other factors controlling water temperature, notably streamflow. Because 2024 was an above‐average water year (period of record: 1909–2024; U.S. Geological Survey 2025) and future summer stream flows are predicted to decrease (Chang et al. 2010), our analysis likely provides a conservative estimate of future water temperature warming.

## 3 Results

### 3.1 Sample Collection, Sequencing, and Quality Control Results

A total of 1947 juvenile samples were collected from the North Umpqua River (2017: *n* = 653; 2018: *n* = 613; 2019: *n* = 681) across 39 locations (Figure 2; Figure S1). These numbers reflect sampling from locations where more than 10 tissue samples were collected; a small number of initially targeted locations were difficult to sample and were subsequently excluded. Of the 1266 samples from 2017 and 2018 that underwent Rapture sequencing, 1227 (97%) had at least 1000 aligned reads and underwent species confirmation analysis. A total of 141 samples (11%) were determined to be salmonid species other than *O. mykiss* , with the majority identified as cutthroat trout (Figure S3; Dataset S1). All but two samples from off‐target species other than cutthroat trout were collected in 2017, indicating improved species identification between years. Off‐target samples were not evenly distributed across sampling sites but showed high prevalence at specific locations (Figure S3). For example, while most locations had no off‐target samples, all samples collected at Steelhead Creek (5c in Figure 2) were cutthroat trout. All off‐target samples were excluded from further analysis, resulting in two locations (Steelhead Creek [5c] and Wright Creek [3d]) with fewer than 10 remaining samples. These two locations were also excluded, leaving 37 locations for analysis. To limit off‐target effects in the 2019 dataset (which did not undergo Rapture sequencing and thus could not be screened for off‐target samples), we excluded seven locations with > 15% cutthroat in 2017 and/or 2018 from the 2019 analyzes (Figure S3).

### 3.2 Geographic Distribution of Run Timing Genotypes

While summer‐run alleles were found at all 37 collection sites in at least one collection year, run timing genotype frequencies showed strong spatial patterns within the basin (Figure 3; Figure S4). The large majority of homozygous summer‐run individuals were observed in Steamboat and Canton Creeks. Among all samples from the 12 Steamboat Creek sites, 84.3% were homozygous summer‐run, 7.2% were heterozygous, and 8.5% were homozygous winter‐run. Notably, all homozygous winter‐run samples originated from the two sampling sites below Steamboat Falls. In addition, a substantially higher winter‐run frequency was observed at the lowest Steamboat Creek location (below Canton Creek; 5a) compared to the next lowest location (above Little Falls; 5b; 46.3% vs. 14.9%). This indicates that Little Falls is not a complete barrier to winter‐run migration, but likely functions as a partial barrier. In contrast, all but one of the 333 samples collected above Steamboat Falls was homozygous summer‐run (the exception was a heterozygote), indicating that Steamboat falls (and/or Black Gorge; see Box 1 and Discussion) is likely a complete barrier to winter‐run steelhead.

Above the falls in Canton Creek, 58.0% were homozygous summer‐run, 21.3% were heterozygous, and 20.8% were homozygous winter‐run. Below the falls, the homozygous summer‐run frequency was 23.8% and the homozygous winter‐run frequency was 33.3%. This indicates that, like Little Falls, Canton Creek Falls is not a complete barrier to winter‐run steelhead but may still impede their access to an extent.

Elsewhere in the basin, homozygous winter‐run was the predominant genotype at nearly all other locations, including above Copeland Creek Falls (6f). An exception was the Upper Fish Creek (7b) site where the summer‐run allele frequency was 97.3%, providing evidence that Fish Creek Falls is an effective barrier to winter‐run steelhead. However, our genetic population structure analysis suggests this is the result of historical rather than contemporary summer‐run steelhead utilization of upper Fish Creek, with upper Fish Creek population likely comprised of stream residents (non‐anadromous fish) with summer‐run ancestry (see below). The highest frequencies of homozygous summer‐run outside Steamboat, Canton, and Fish creeks occurred at one mainstem location just upstream of the Steamboat Creek confluence, at Rock Creek sites upstream of the Rock Creek summer‐run hatchery program, and at one location high in Little River. Thus, under current conditions, Steamboat Falls (and/or Black Gorge; see Box 1 and Discussion) and Fish Creek Falls are the only barriers that effectively exclude winter‐run access to fish‐bearing streams under current conditions in the North Umpqua basin.

### 3.3 Variability of Allele Frequencies Between Years

**FIGURE 4:**
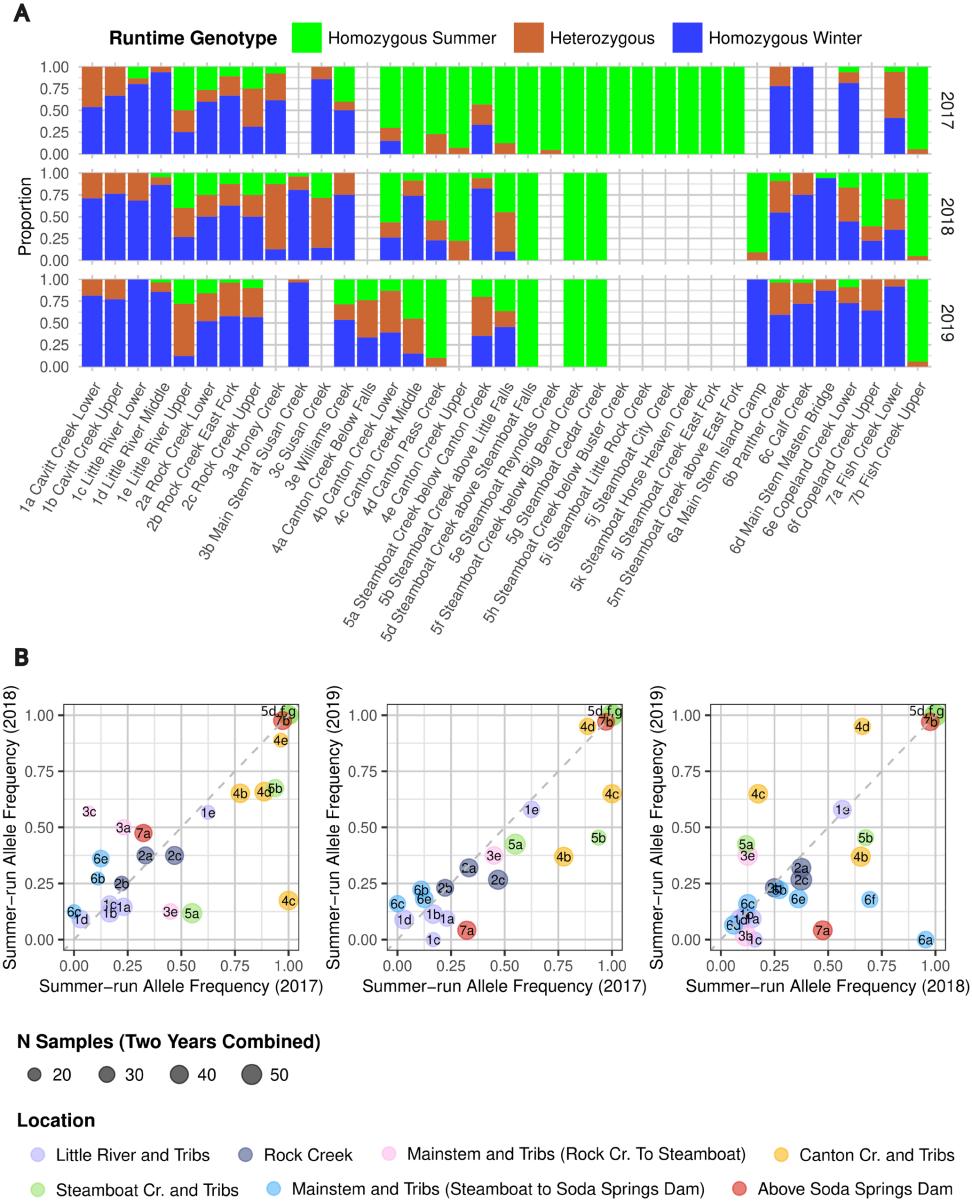
*GREB1L* genotypes and allele frequencies by year. (A) Run timing genotype proportions among samples from each collection location and year; (B) comparison of summer‐run allele frequencies between years for each location. Circle color indicates general sample collection area, labels indicate precise sampling location for each point as indicated in part A and Figure 2. Circle size indicates the total number of samples collected in the two comparison years. The diagonal dashed line indicates the position of points if allele frequencies are identical between comparison years.

To determine the stability in patterns of run timing distribution between years, we explored the allele frequencies between sample years and sample sites. Across the full sample set, the mean allele frequencies of the summer‐run variant were 0.464 in 2017, 0.454 in 2018, and 0.581 in 2019. We then compared summer‐run allele frequencies at each sampling site across years through pairwise comparisons (Figure 4). Allele and genotype frequencies were generally similar between years for a given location (Figure 4; Figure S4). The most notable exceptions were locations in Canton Creek above the falls and the two lower locations in Steamboat Creek (both below Steamboat Falls) (Figure 4; Figure S4), suggesting the ability of winter‐run steelhead to migrate above Canton Creek Falls and Little Falls may vary between years. The mainstem at Island Camp (6a) also exhibited large differences in summer‐run frequencies between years. Island Camp is located just above the confluence with Steamboat Creek, and therefore this discrepancy may represent differences in the dispersion patterns of juvenile summer‐run steelhead exiting Steamboat Creek. The locations with the least variability in allele frequencies were located above Steamboat Falls and above Fish Creek Falls, where summer‐run allele frequencies were close to 100% in all years.

### 3.4 Population Structure of North Umpqua *O. mykiss*

**FIGURE 5:**
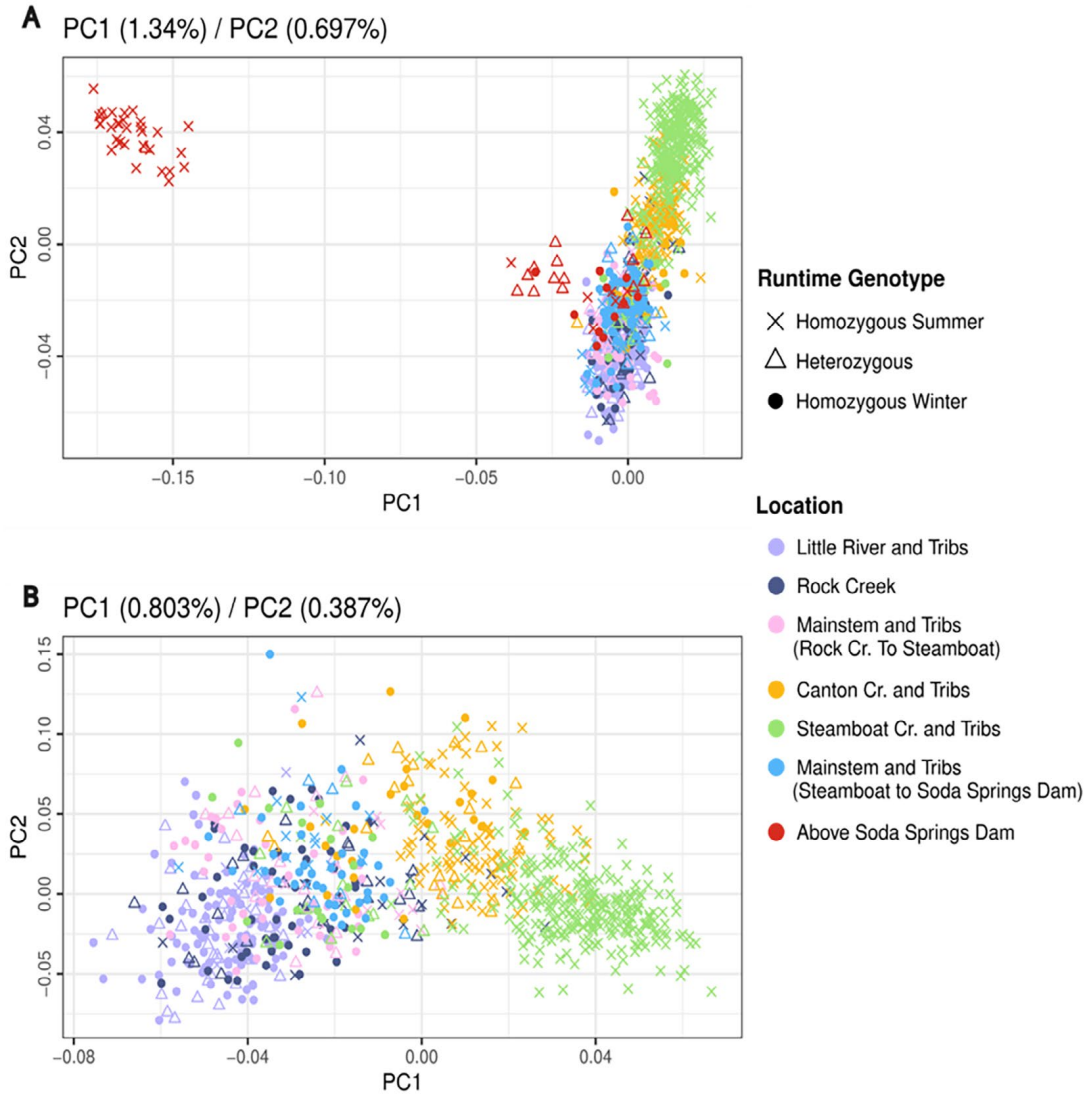
Population structure of North Umpqua juvenile *O. mykiss* and relationship with run timing. (A) Principal Component Analysis (PCA) with all sampling locations included. Red samples on the left side of the plot (PC1 < −0.10) were collected at the (7b) Fish Creek Upper site; red samples on the right side of the plot (PC1 > −0.05) were collected at the (7a) Fish Creek Lower site; (B) PCA of all locations excluding Fish Creek and Copeland Creek sites. See also Figure S5.

A total of 12,689 SNPs passed filtering for inclusion in the genetic population structure analysis. The first axis of the PCA revealed that individuals from the Fish Creek Upper location (7b) were the most genetically distinct group in the dataset (Figure 5A). Some samples from the lower Fish Creek site (7a) were also distinct, but to a lesser extent. Fish Creek was isolated from the rest of the basin by Soda Springs Dam for 60 years, so a degree of distinctness due to genetic drift is expected (see Discussion also). The second PCA axis suggested a relationship between run timing and population structure in the basin. After removal of Fish Creek samples, the first axis indicated genetic population structure related to geography and run timing. Samples from upper Copeland Creek (6f) separated from samples in the rest of the basin along PC2, but with a much lower percentage of variance explained compared to PC1 (Figure S5), indicating upper Copeland Creek samples were not substantially distinct from the rest of the basin.

To further clarify the genetic population structure relationships in the rest of the basin, we reran the PCA with both Fish Creek and Copeland Creek samples excluded. The resulting PCA clearly showed genetic differentiation existing on a gradient between locations with higher summer‐run allele frequencies (Steamboat and Canton Creeks) and lower summer‐run allele frequencies (Figure 5B). Summer‐run steelhead from Steamboat Creek appear more distinct from populations elsewhere in the basin than do samples from Canton Creek. Homozygous winter‐run steelhead from Steamboat below Steamboat Falls clustered with samples from the mainstem North Umpqua River. While geography may also play a role in population structure, distance does not appear to be the sole driver, as locations higher up on the mainstem than Steamboat and Canton Creeks clustered with lower mainstem locations with a similarly lower summer‐run allele frequency. The results are consistent with Steamboat Falls (and/or Black Gorge) reducing genetic connectivity between summer‐run and winter‐run steelhead, with a greater degree of genetic mixing between runs occurring in Canton Creek. These results indicate that population structure in the North Umpqua basin is associated with both geography and run timing.

### 3.5 Soda Springs Fish Ladder Could Be Closed to the Winter‐Run With Minimal Impacts on Other Species

**FIGURE 6:**
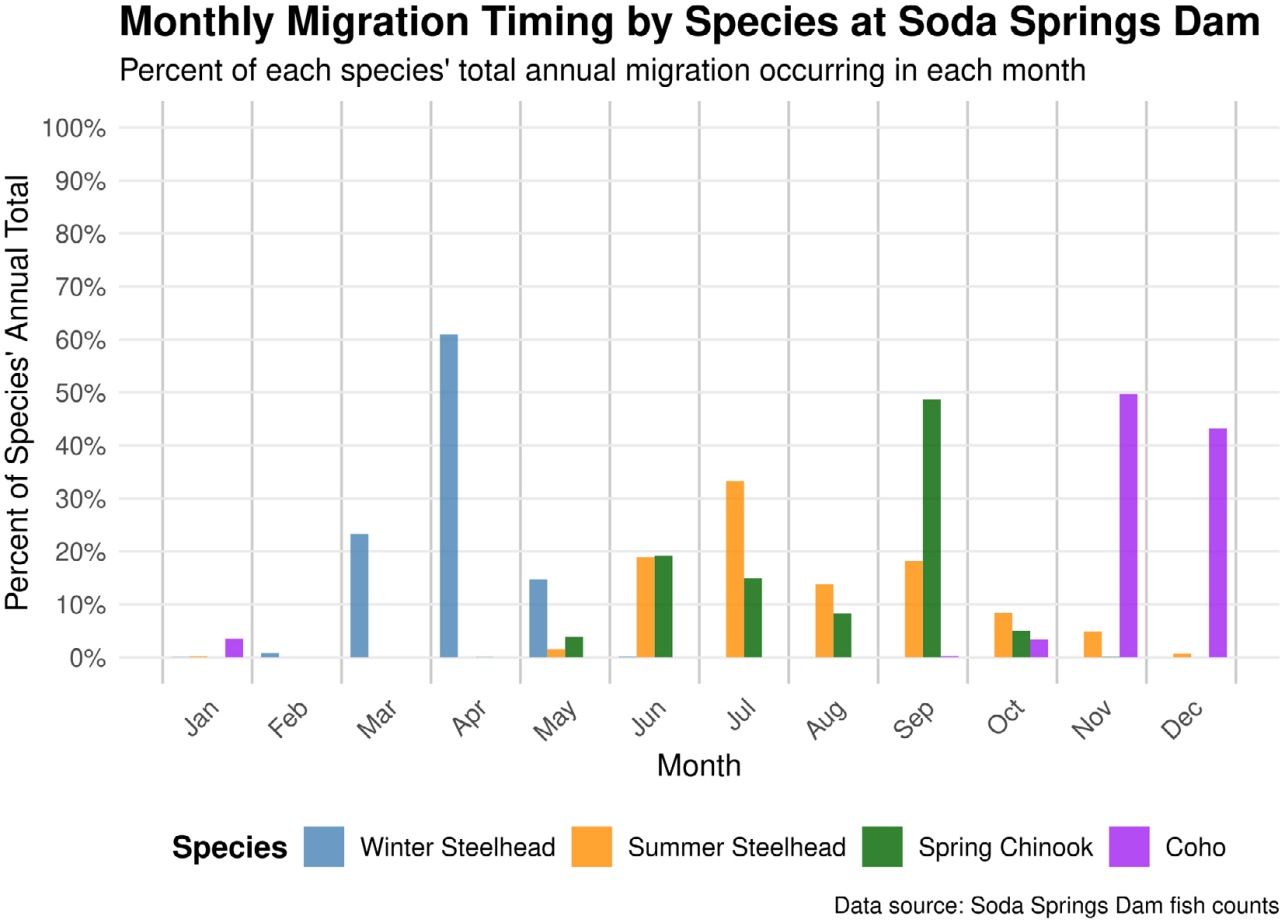
Species passing Soda Springs Fish Ladder by date (2013–2019).

Our analysis of the timing of anadromous species passing through Soda Springs fish ladder indicated that there is very little overlap in the timing of winter‐run steelhead passage and that of other anadromous species (Figure 6). The large majority of winter‐run steelhead pass the fish ladder between February and the end of April, and their passage is complete by mid‐May. A small proportion (< 5%) of summer‐run steelhead and spring‐run Chinook salmon pass the ladder in May, mainly toward the end of the month, before their numbers increase in June. No Pacific lamprey were observed passing the ladder in this dataset, but recolonization of that species above the dam is a management goal. Lamprey passage would be expected to begin at a similar time to spring‐run Chinook salmon. Thus, closing the ladder from February through mid‐May would exclude most winter‐run steelhead from the area above Soda Springs Dam while having minimal interference with the passage of other anadromous species.

Our analysis of the potential impact of such an action on winter‐run steelhead indicated that closing the Soda Springs fish ladder between February and early May would have only a minor impact on the total winter‐run steelhead population. In the years analyzed (2013–2024), between 139 and 375 wild adult winter‐run steelhead were observed passing the Soda Springs fish ladder each year. These numbers represent 2.4%–4.5% of the total estimates for wild adult winter‐run steelhead passing Winchester Dam in the lower North Umpqua (Figure 1) in each of those years, respectively. In other words, > 95% of the winter‐run population would be unaffected by closure of the fish ladder. These results indicate that only a very small proportion of the winter‐run steelhead population is currently using the habitat above Soda Springs Dam, and their exclusion from that habitat would minimally impact the overall winter‐run steelhead population.

### 3.6 Current Summer‐Run Habitat Is Vulnerable to Climate Change

**FIGURE 7:**
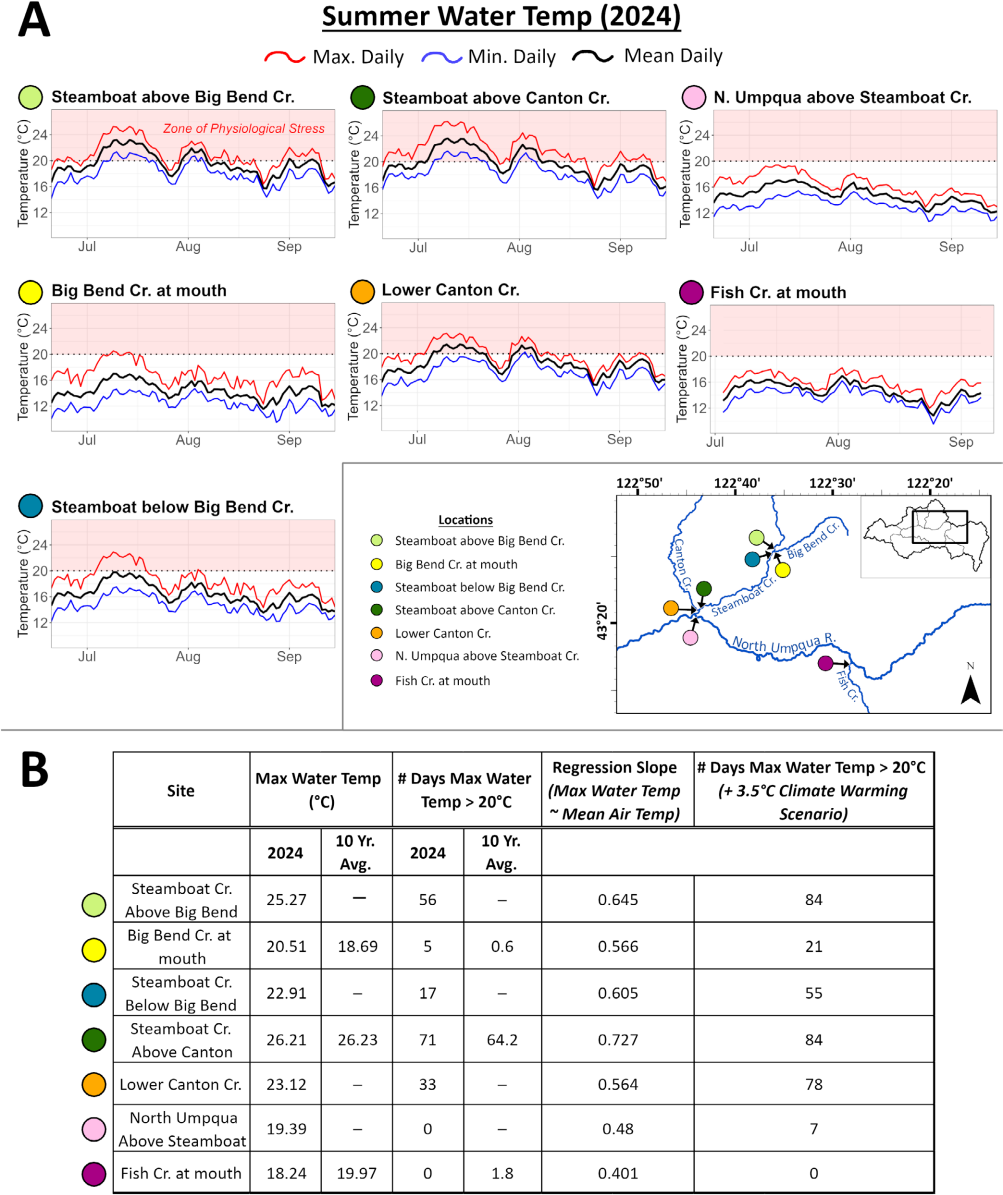
Water temperature data and climate scenario analyzes. (A) Time series of mean, minimum, and maximum daily water temperature at seven sites during the 2024 summer period. (B) Single‐year (2024) and 10‐year average temperature metrics (where available); the final column shows the projected number of days with water temperature > 20°C under a likely air‐temperature warming scenario, based on a regression fitted to 2024 empirical data.

Our analysis revealed that maximum water temperatures frequently exceeded the 20°C temperature threshold for physiological stress in Steamboat and Canton creeks, the streams where juvenile steelhead genotypes were either predominately or entirely summer‐run (Figures 3 and 7). Big Bend Creek, a major cold‐water tributary to Steamboat Creek, exceeded 20°C on only 5 days in 2024 (Figure 7) and was sufficiently cool to reduce Steamboat Creek MDMT by nearly 3°C, and to reduce the number of days Steamboat Creek exceeded 20°C from 53 days to 17 days at sites immediately above and below the confluence with Big Bend Creek, respectively (Figure 7). Below Big Bend Creek, Steamboat Creek warmed significantly. At its confluence with Canton Creek (17 km downstream), MDMT was > 26°C and exceeded 20°C for 71 days in 2024 (Figure 7). Unlike Big Bend Creek, Canton Creek was only slightly cooler than Steamboat Creek. Lower Canton Creek MDMT was > 23°C and temperatures exceeded 20°C on 33 days in 2024 (Figure 7). The confluence with Canton Creek had a minor cooling effect on Steamboat Creek, reducing MDMT by approximately 1°C and reducing the number of days exceeding 20°C from 71 to 54 in Steamboat Creek immediately above and below Canton Creek, respectively (Figure S6). In contrast to Steamboat and Canton creeks, sites in the North Umpqua mainstem immediately above and below Steamboat Creek and at the mouth of Fish Creek never exceeded 20°C in 2024 (MDMT were 19.4°C and 18.2°C, respectively; Figure S6).

Our modeling of stream temperature responses to an air temperature increase of 3.5°C estimated a substantial increase in the number of days with water temperatures exceeding 20°C at all sites except for Fish creek (Figure 7). Under the 3.5°C air temperature warming scenario, Steamboat Creek below Big Bend Creek had 55 days exceeding 20°C (as compared to 17 days observed in 2024). Big Bend Creek itself was estimated to increase from 5 to 21 days exceeding 20°C under the warming scenario. The North Umpqua mainstem at Steamboat Creek had an increase from 0 to 7 days exceeding 20°C under the warming model, indicating the mainstem may be relatively buffered from climate warming impacts, but is still predicted to periodically warm to physiologically stressful levels in the future. Fish Creek was the only site where temperatures never exceeded 20°C when the 3.5°C increase was applied to the 2024 observations. Fish Creek averaged 1.8 days exceeding 20°C over the past 10 years, and thus stressful conditions occasionally occur in Fish Creek and may increase with climate warming. However, the slope of the regression between maximum water temperature and mean air temperature at Fish Creek was the lowest of any temperature measurement sites (Figure 7; Figure S6), indicating that water temperatures in Fish Creek are predicted to have the lowest rate of stream warming for a given increase in air temperature.

## 4 Discussion

The North Umpqua River is one of the last strongholds for summer‐run steelhead in the coastal rivers of the West Coast of North America. However, unprecedented low returns and fisheries closures during the past decade have raised conservation concerns about the resilience of this population (ODFW 2022, 2023). Given the critical importance of exclusive habitat for summer‐run population persistence (Quinn et al. 2016; Kannry et al. 2020; Waples et al. 2022), we applied genetic tools to map the distribution of summer‐run steelhead juveniles throughout the basin and better characterize life history tradeoffs associated with steelhead run timing, including the relationship with putative flow‐dependent barriers and stream temperature. Our analysis revealed that among the many natural cascades and falls within the basin below Soda Springs Dam, Steamboat Falls (and/or Black Gorge) is the only barrier confirmed to currently exclude winter‐run steelhead while allowing summer‐run steelhead passage. We then explored the feasibility of expanding exclusive summer‐run steelhead habitat in the basin by operating the Soda Springs Dam fish ladder to selectively pass summer‐run steelhead into upstream habitat and exclude winter‐run steelhead. Our analysis indicated that such management could be implemented with minimal impacts to other anadromous species and the winter‐run steelhead population. Questions remain about the full extent of exclusive habitat such management could create, which would be much greater if Fish Creek Falls is passable to summer‐run steelhead, but which would still be significant if it is not passable. Finally, we assessed the vulnerability of current and potential summer‐run steelhead habitat to warming above thermally stressful levels for steelhead. Our results demonstrate that contemporary summer‐run steelhead habitat (i.e., Steamboat Creek above Steamboat Falls), including the most important oversummering pool for adults in Steamboat Creek, already experiences periods of stressful temperatures, with the frequency of such conditions projected to increase substantially under late‐century climate scenarios. In contrast, suitable summer‐run steelhead habitat above Soda Springs Dam appears more buffered from warming than other areas in the basin.

The summer‐run steelhead life history incurs substantial costs relative to winter‐run steelhead, including reduced body size and fecundity, the metabolic strain of holding and maturing while fasting in freshwater, and prolonged exposure to environmental stress and predation within the confined riverine environment. Selection against the summer‐run steelhead life history should eliminate it from a basin unless it confers compensating advantages. In other words, summer‐run steelhead are expected to persist only in rivers where they possess a clear evolutionary advantage. Both theoretical frameworks and empirical evidence (including from this study) indicate that access to exclusive or near‐exclusive spawning and rearing habitat is the primary benefit of the summer‐run life history (Quinn et al. 2016; Kannry et al. 2020; Waples et al. 2022). For example, Quinn et al. (2016) hypothesized that physical factors creating temporally limited habitat accessibility can be the primary driver of early migration timing. Withler (1966) observed that British Columbia, CA summer‐run steelhead populations are only found in watersheds with natural seasonal barriers. In another empirical example, Kannry et al. (2020) mapped the distribution of summer‐ and winter‐run steelhead alleles in the Eel River, California, USA and identified flow‐dependent barriers as strong breakpoints between runs. Access to exclusive habitat also drives early migration timing in other salmonid species, including Chinook and coho salmon (Sandercock 1991; Quinn et al. 2016; Thompson et al. 2019; Waples et al. 2022).

In the North Umpqua basin, our findings indicate that Steamboat Creek above Steamboat Falls represents the only habitat that can be considered exclusive to summer‐run steelhead use. The low frequencies of summer‐run juveniles and alleles observed in most other locations likely result from straying from the Steamboat Creek population and/or dispersal from Rock Creek Hatchery. This interpretation is supported by our population structure analysis, where five juveniles homozygous for the summer‐run allele collected in Rock Creek clustered with Canton Creek and Steamboat Creek samples in the PCA (Figure 5), suggesting that they had one or more parents who strayed (or were taken as broodstock) from the Steamboat Creek basin. The ability of winter‐run steelhead to pass above Canton Creek Falls and the substantial interannual variation in genotype and allele frequencies observed at many Canton Creek locations (Figure 4) also suggest that Canton Creek summer‐run steelhead are supported, at least to some extent, by ongoing spillover or straying from the population above Steamboat Falls, and may not represent a fully self‐sustaining population. These findings underscore the critical dependence of North Umpqua summer‐run steelhead on a single exclusive spawning area, with the population above Steamboat Falls supporting summer‐run presence throughout the broader basin.

Populations that can utilize a diversity of habitats are buffered against environmental disturbances within individual habitat patches (Brennan et al. 2019). Conversely, limited habitat diversity reduces population resilience and increases vulnerability to local disturbances. The role of Steamboat Creek as the sole area of exclusive summer‐run steelhead habitat in the North Umpqua basin exemplifies this vulnerability. For example, Big Bend Creek provides the cold‐water inputs that maintain the most important oversummering pool in Steamboat Creek, but wildfire burned extensive areas of the Big Bend Creek watershed in 2021. The resulting loss of riparian shading may have contributed to the elevated number of days in 2024 when water temperatures at the mouth of Big Bend Creek exceeded 20°C relative to the 10‐year average. This represents just one of multiple large wildfires that have affected the Steamboat Creek watershed over the past decade (National Interagency Fire Center (NIFC) 2025). Our temperature analyzes demonstrate that oversummering habitat in Steamboat Creek already exposes adult summer‐run steelhead to thermally stressful conditions (17 days exceeding 20°C directly below the primary holding pool in 2024), which will substantially increase under projected climate warming. Temperatures of this magnitude cause physiological stress and migration delays for adult steelhead (Keefer et al. 2018) and decrease the extent of thermally suitable rearing habitat for juvenile steelhead who face competitive exclusion by more warm‐tolerant species (Reeves et al. 1987). Thus, the concentration of the summer‐run steelhead spawning population within a single, increasingly stressed watershed creates an urgent conservation concern and underscores the need for habitat diversification to ensure long‐term population persistence.

Although Steamboat Creek's role as the sole source of exclusive summer‐run steelhead habitat creates vulnerabilities, some diversity in habitat use is expressed by oversummering adults. A portion of the summer‐run steelhead population over‐summers in the mainstem North Umpqua near the mouth of Steamboat Creek before moving to spawning areas in Steamboat Creek, Canton Creek, and other tributaries later in the year (Loomis et al. 2003). This mainstem holding strategy provides access to relatively cool thermal refuges, but it also entails significant tradeoffs. Unlike Steamboat Creek, where recreational fishing is prohibited, mainstem holding areas experience intensive angling pressure. Although the fishery operates under catch‐and‐release and other restrictive regulations designed to protect fish, handling mortality is inevitable, and the cumulative stress from repeated captures throughout the summer may rival or exceed the physiological stress imposed by temperatures above 20°C (Bentley 2017; Twardek et al. 2018). Perhaps the most critical risk of mainstem holding is the potential for fish to miss the optimal timing window for ascending to exclusive spawning habitat (e.g., between the first fall rains in September/October and when flows become substantially higher, typically in November). Fish that fail to ascend Steamboat Falls would be forced to spawn in areas accessible to winter‐run steelhead, thereby facing direct reproductive competition with larger, more fecund, and more abundant winter‐run counterparts. While this diversity in holding strategies likely provides some population‐level buffering against localized disturbances, it does not address the fundamental constraint that exclusive spawning habitat remains restricted to the area above Steamboat Falls, leaving the reproductive core of the population concentrated in a single vulnerable watershed.

Operating the Soda Springs Dam fish ladder as an artificial flow‐dependent barrier presents a rare management opportunity to address summer‐run steelhead conservation needs at the most fundamental level by expanding exclusive spawning and rearing habitat. Our analysis indicates that such management could be implemented with minimal impacts on other anadromous species, including winter‐run steelhead. Greater than 95% of the winter‐run steelhead population—which far exceeds summer‐run steelhead in abundance—currently spawns downstream of Soda Springs Dam. The small proportion of winter‐run steelhead that would be prevented from passing upstream would likely spawn near the dam (Quinones et al. 2015), which may result in localized density dependent effects on juvenile growth and survival, but which would likely have minimal population‐level effects. Nevertheless, we suggest that management of passage at Soda Springs could be monitored and adaptively managed. Without seasonal ladder closure, the habitat above Soda Springs Dam will continue to serve primarily winter‐run steelhead, as evidenced by the 76%–96% (median = 86%) of wild steelhead passing the ladder annually between 2012 and 2024 that were winter‐run.

Expanding exclusive summer‐run steelhead habitat to the area above Soda Springs Dam would provide three primary conservation benefits. First, it would increase the overall abundance of summer‐run steelhead in the North Umpqua basin. While the precise production capacity of this habitat remains uncertain, at least 11 km of suitable spawning habitat exists above the dam. Although 174–441 wild steelhead have passed the ladder annually since its installation, full habitat productivity may not yet be realized given that steelhead recolonization can require multiple generations and proceed more slowly than in other salmonid species (Weigel et al. 2013). Second, creating a spatially distinct area of exclusive spawning habitat would address the current “all eggs in one basket” vulnerability, wherein the overwhelming majority of summer‐run steelhead production depends on Steamboat Creek. The habitat above Soda Springs Dam, including Fish Creek, is geographically separated from Steamboat Creek and more resilient to climate warming due to its higher elevation and greater groundwater contribution to streamflows. Third, establishing exclusive summer‐run steelhead habitat above the dam could facilitate recolonization of upper Fish Creek, which contains 50–65 km of high‐elevation, cold‐water habitat. This represents perhaps the most substantial long‐term benefit, though the recolonization potential of this stream remains less certain.

Two lines of evidence suggest summer‐run steelhead historically used habitat in upper Fish Creek. First, upper Fish Creek exhibited the highest frequencies of summer‐run steelhead alleles of any location outside Steamboat Creek (Figures 3 and 4), strongly indicating that any historical steelhead in this area were summer‐run and left genetic signatures that persist in the current, primarily resident population. Second, early USFS records reportedly documented steelhead observations at a USFS station above Fish Creek Falls in the early 1900s, when rangers maintained daily operational diaries (Coates 2012). Our population structure analysis revealed that upper Fish Creek samples were the most genetically distinct group in the dataset (Figure 5A), which could reflect either long‐term isolation as resident rainbow trout or genetic drift acting on an already distinct anadromous population following dam‐induced isolation. A historical summer‐run steelhead population in upper Fish Creek would likely have been distinct from fish below Fish Creek Falls, similar to how summer‐run steelhead above Steamboat Falls are genetically distinct from winter‐run steelhead in the mainstem. Given the geographic separation, upper Fish Creek would probably have differed from the Steamboat Creek summer‐run population as well. Regardless, the genetic distinctiveness observed in our samples suggests that steelhead had not yet reascended Fish Creek Falls prior to our sampling period.

Natural recolonization of upper Fish Creek would likely be a gradual process due to the formidable nature of Fish Creek Falls, which probably presents a greater challenge than Steamboat Falls or any other passable barrier in the basin. The obstacle consists of over a kilometer of multiple waterfalls and high‐velocity sections that would challenge fish passage even under optimal low‐flow conditions. Without strong natal homing instincts to serve as motivation, the likelihood of attempted passage may be low, suggesting that a substantial population of summer‐run steelhead in the habitat above Soda Springs Dam may be necessary to generate enough individuals willing to attempt recolonization. Alternatively, assisted translocation of summer steelhead above the falls could be considered to re‐establish anadromy in upper Fish Creek. Despite these challenges, successful recolonization would provide access to 50–65 km of high‐elevation, cold‐water habitat that could substantially enhance the North Umpqua's summer‐run steelhead long‐term resilience and productivity. This underscores the importance of establishing a robust summer‐run population above Soda Springs Dam to promote successful expansion into the upper Fish Creek system.

While excluding winter‐run steelhead from habitat to benefit summer‐run steelhead represents a relatively novel management approach, there is an extensive history of actions that have destroyed or degraded early‐run (e.g., summer steelhead, spring Chinook) habitat exclusivity across multiple salmonid species, often undertaken explicitly to benefit late‐run populations (e.g., winter steelhead, fall Chinook) without consideration of the impacts on the early‐run life histories. Many dams have altered temperature and flow regimes or provided fish ladders that facilitate late‐run access to previously exclusive early‐run habitat (Thompson et al. 2019; Waples et al. 2022). Similarly, numerous watersheds have histories of flow‐dependent barriers being modified with explosives or fish ladders to enhance late‐run passage (Waples et al. 2022). An example is the Siletz River, Oregon, USA: a fish ladder installed in the 1950s to facilitate passage over a flow‐dependent barrier enabled winter‐run colonization of previously exclusive summer‐run habitat, leading to summer‐run declines (Hemstrom et al. 2018). Managers subsequently added a trap to the ladder in the 1990s and now operate it to exclude winter‐run steelhead, effectively recreating habitat exclusivity for the summer‐run steelhead population. Notably, such barrier modifications have typically proceeded without consideration of consequences for summer‐run steelhead populations. Even within the North Umpqua, a fish ladder was constructed at Steamboat Falls to facilitate year‐round fish passage. However, our results indicate that either the design was insufficient (e.g., the poor design originally resulted in the ladder becoming clogged with debris in the winter, although this issue was reportedly fixed with later modifications), or the falls and constrictions in nearby Black Gorge are the actual barrier to winter‐run migration. Unlike permanent infrastructure modifications such as dams and barrier removal, seasonal operation of the existing Soda Springs fish ladder represents a reversible management tool that could be modified or suspended annually if unforeseen consequences warranted reevaluation, making it an ideal candidate for adaptive management of summer‐run steelhead habitat exclusivity.

In conclusion, this study demonstrates that conserving intraspecific diversity under climate change will require management strategies that explicitly recognize and preserve the ecological processes sustaining different life histories. For summer‐run steelhead, access to exclusive spawning and rearing habitat is not simply beneficial but foundational to the persistence of the life history itself. Our findings show that this diversity can become precariously dependent on a single watershed and a small number of thermal refuges, creating acute vulnerability to warming temperatures, wildfire, altered hydrology, and other climate‐driven disturbances. At the same time, the study illustrates that targeted management interventions can meaningfully improve resilience. By identifying the specific barriers, habitats, and thermal conditions that maintain summer‐run steelhead, genomic tools provide managers with the ability to move beyond generalized conservation approaches and instead protect the ecological mechanisms that generate and sustain adaptive diversity.

More broadly, the North Umpqua case study highlights how climate adaptation for migratory fishes may increasingly depend on restoring or strategically managing habitat heterogeneity across river networks. Expanding access to cold‐water refuges, reconnecting climate‐resilient headwater habitats, and preserving spatially distinct spawning areas can reduce the risks associated with concentrating populations into a single vulnerable habitat patch. In some cases, this may require reconsidering historical management paradigms that prioritized maximizing overall fish passage without accounting for the evolutionary importance of selective barriers and temporally partitioned habitat use. As climate change accelerates, conservation strategies that maintain life history diversity will likely be critical not only for summer‐run steelhead but for migratory fish populations worldwide whose resilience depends on the continued expression of diverse, locally adapted phenotypes. Protecting these forms of diversity is ultimately essential for sustaining the ecological, evolutionary, cultural, and economic values that salmonid populations provide in an increasingly uncertain future.

## Funding

This work was supported by North Umpqua Foundation. U.S. Forest Service.

## Conflicts of Interest

The authors declare no conflicts of interest.

## Supporting information


**Table S1:** Species validation sample information. Data from the samples listed in the table were downloaded from NCBI and used as known‐species samples in the principal component analysis used to identify off‐target juvenile samples collected in the North Umpqua.
**Table S2:** Primer sequence information for the qPCR assay for steelhead run timing genotype.
**Figure S1:** All juvenile samples collected from the North Umpqua in 2017, 2018, and 2019, shown by collection location. Off‐target samples have not been removed; (A) all years combined; (B) samples divided by collection year.
**Figure S2:** Species confirmation analyzes. Principal component analysis of all juvenile salmonids collected in the North Umpqua as part of this study combined with data from known species listed in Table S1. Samples were considered to be *O. mykiss* if at a negative position on both PC1 and PC2 (the tight lower left cluster). Cutthroat were defined as samples positioned in the positive numbers along PC2 (this likely includes some steelhead/cutthroat hybrids). Chinook and Coho salmon are the clusters to the right of 0.050 on PC1 and below 0.00 on PC2.
**Figure S3:** Proportion of off‐target species at each location in 2017 and 2018; (A) includes all off‐target species; (B) off‐target species other than cutthroat have been excluded. Dashed line indicates 15% cutthroat cutoff for location inclusion in 2019 dataset.
**Figure S4:** Stacked bar graph counts of run timing genotypes at each location and year.
**Figure S5:** Principal Component Analysis of North Umpqua juveniles. All locations included except Fish Creek sites. Blue‐colored outliers along PC2 are samples from the (6f) Copeland Creek Upper site.
**Figure S6:** Water to air temperature regression analyzes.

**Dataset: S1.** Individual fish genetic sample details, including the year and location of sampling, and individual fish length and genetics data.

## Data Availability

All sequencing data generated in this project are available on NCBI Sequence Read Archive under BioProject ID PRJNA1476309 [http://www.ncbi.nlm.nih.gov/bioproject/1476309]. Primer and probe sequences for the qPCR assay used in this study, qPCR genotyping results for each sample, and individual sample attributes are available in the Supporting Information.

## References

[eva70293-bib-0001] Ali, O. A. , S. M. O’Rourke , S. J. Amish , et al. 2016. “RAD Capture (Rapture): Flexible and Efficient Sequence‐Based Genotyping.” Genetics 202, no. 2: 389–400. 10.1534/genetics.115.183665. 26715661 PMC4788223

[eva70293-bib-0002] Baigún, C. R. M. 2003. “Characteristics of Deep Pools Used by Adult Summer Steelhead in Steamboat Creek, Oregon.” North American Journal of Fisheries Management 23, no. 4: 1167–1174. 10.1577/M02-001.

[eva70293-bib-0003] Baird, N. A. , P. D. Etter , T. S. Atwood , et al. 2008. “Rapid SNP Discovery and Genetic Mapping Using Sequenced RAD Markers.” PLoS One 3, no. 10: e3376. 10.1371/journal.pone.0003376. 18852878 PMC2557064

[eva70293-bib-0004] Barker, T. 2021. “B.C. Closes Skeena Watershed for Steelhead Effective Oct 12.” The Northern View, 7 October. Accessed October 19, 2025. https://www.thenorthernview.com/news/b‐c‐closes‐skeena‐watershed‐for‐steelhead‐effective‐oct‐12‐5986300.

[eva70293-bib-0005] Bentley, K. T. 2017. Evaluation of Creel Survey Methodology for Steelhead Fisheries on the Quillayute and Hoh Rivers. Washington Department of Fish and Wildlife.

[eva70293-bib-0006] Blumm, M. C. , and B. Kloos . 1986. “Small Scale Hydropower and Anadromous Fish: Lessons and Questions From the Winchester Dam Controversy.” In Symposium on Salmon Law, 583–637. Lewis & Clark Law School. https://www.jstor.org/stable/43265767.

[eva70293-bib-0007] Braun, D. C. , J. W. Moore , J. Candy , and R. E. Bailey . 2016. “Population Diversity in Salmon: Linkages Among Response, Genetic and Life History Diversity.” Ecography 39, no. 3: 317–328. 10.1111/ecog.01102.

[eva70293-bib-0008] Brennan, S. R. , D. E. Schindler , T. J. Cline , T. E. Walsworth , G. Buck , and D. P. Fernandez . 2019. “Shifting Habitat Mosaics and Fish Production Across River Basins.” Science 364, no. 6442: 783–786. 10.1126/science.aav4313. 31123135

[eva70293-bib-0009] Busby, P. J. , T. C. Wainwright , G. J. Bryant , et al. 1996. “Status Review of West Coast Steelhead From Washington, Idaho, Oregon, and California.” In NOAA Technical Memorandum NMFS‐NWFSC‐27. National Marine Fisheries Service.

[eva70293-bib-0010] Carlson, S. M. , and W. H. Satterthwaite . 2011. “Weakened Portfolio Effect in a Collapsed Salmon Population Complex.” Canadian Journal of Fisheries and Aquatic Sciences 68, no. 9: 1579–1589. 10.1139/f2011-084.

[eva70293-bib-0063] Chang, H. , J. Jones , M. Gannett , et al. 2010. “Climate Change and Fresh Water Resources in Oregon.” In Oregon Climate Change Research Institute, Oregon Climate Assessment Report, edited by K. D. Dello and P. W. Mote . College of Oceanic and Atmospheric Sciences, Oregon State University.

[eva70293-bib-0064] Coates, K. C. 2012. History, Ecology and Restoration Potential of Salmonid Fishes in the Umpqua River, Oregon. Master's Thesis, 519. University of Montana.

[eva70293-bib-0011] Danecek, P. , J. K. Bonfield , J. Liddle , et al. 2021. “Twelve Years of SAMtools and BCFtools.” GigaScience 10, no. 2: giab008. 10.1093/gigascience/giab008. 33590861 PMC7931819

[eva70293-bib-0012] Des Roches, S. , L. H. Pendleton , B. Shapiro , and E. P. Palkovacs . 2021. “Conserving Intraspecific Variation for Nature's Contributions to People.” Nature Ecology & Evolution 5, no. 5: 574–582. 10.1038/s41559-021-01403-5. 33649544

[eva70293-bib-0013] Des Roches, S. , D. M. Post , N. E. Turley , et al. 2018. “The Ecological Importance of Intraspecific Variation.” Nature Ecology & Evolution 2, no. 1: 57–64. 10.1038/s41559-017-0402-5. 29203921

[eva70293-bib-0014] Dressler, T. L. , K. Anlauf‐Dunn , A. Chandler , and E. J. Eliason . 2025. “Beyond Latitude: Thermal Tolerance and Vulnerability of a Broadly Distributed Salmonid Across a Habitat Temperature Gradient.” Conservation Physiology 13, no. 1: coaf030. 10.1093/conphys/coaf030. 40313657 PMC12043440

[eva70293-bib-0015] Fleishman, E. 2025. Seventh Oregon Climate Assessment. Oregon Climate Change Research Institute: Oregon State University. 10.5399/osu/1181.

[eva70293-bib-0016] Fraik, A. K. , J. R. McMillan , M. Liermann , et al. 2021. “The Impacts of Dam Construction and Removal on the Genetics of Recovering Steelhead ( *Oncorhynchus mykiss* ) Populations Across the Elwha River Watershed.” Genes 12, no. 1: 89. 33450806 10.3390/genes12010089 PMC7828262

[eva70293-bib-0017] Greene, C. M. , J. E. Hall , K. R. Guilbault , and T. P. Quinn . 2009. “Improved Viability of Populations With Diverse Life‐History Portfolios.” Biology Letters 6, no. 3: 382–386. 10.1098/rsbl.2009.0780. 20007162 PMC2880035

[eva70293-bib-0018] Hemstrom, W. , S. van de Wetering , and M. Banks . 2018. “Fish Ladder Installation Across a Historical Barrier Asymmetrically Increased Conspecific Introgressive Hybridization Between Wild Winter and Summer Run Steelhead Salmon in the Siletz River, Oregon.” Canadian Journal of Fisheries and Aquatic Sciences 75, no. 9: 1383–1392. 10.1139/cjfas-2016-0411.

[eva70293-bib-0019] Hess, J. E. , J. S. Zendt , A. R. Matala , and S. R. Narum . 2016. “Genetic Basis of Adult Migration Timing in Anadromous Steelhead Discovered Through Multivariate Association Testing.” Proceedings. Biological Sciences 283, no. 1830: 20153064. 27170720 10.1098/rspb.2015.3064 PMC4874702

[eva70293-bib-0020] Hilborn, R. , T. P. Quinn , D. E. Schindler , and D. E. Rogers . 2003. “Biocomplexity and Fisheries Sustainability.” Proceedings of the National Academy of Sciences 100, no. 11: 6564–6568. 10.1073/pnas.1037274100. PMC164486 12743372

[eva70293-bib-0021] Kannry, S. H. , S. M. O’Rourke , S. J. Kelson , and M. R. Miller . 2020. “On the Ecology and Distribution of Steelhead ( *Oncorhynchus mykiss* ) in California's Eel River.” Journal of Heredity 111, no. 6: 548–563. 33125465 10.1093/jhered/esaa043

[eva70293-bib-0022] Keefer, M. L. , T. S. Clabough , M. A. Jepson , E. L. Johnson , C. A. Peery , and C. C. Caudill . 2018. “Thermal Exposure of Adult Chinook Salmon and Steelhead: Diverse Behavioral Strategies in a Large and Warming River System.” PLoS One 13, no. 9: e0204274. 10.1371/journal.pone.0204274. 30240404 PMC6150539

[eva70293-bib-0023] Keefer, M. L. , C. A. Peery , and B. High . 2009. “Behavioral Thermoregulation and Associated Mortality Trade‐Offs in Migrating Adult Steelhead ( *Oncorhynchus mykiss* ): Variability Among Sympatric Populations.” Canadian Journal of Fisheries and Aquatic Sciences 66, no. 10: 1734–1747. 10.1139/F09-131.

[eva70293-bib-0024] Kim, S. Y. , K. E. Lohmueller , A. Albrechtsen , et al. 2011. “Estimation of Allele Frequency and Association Mapping Using Next‐Generation Sequencing Data.” BMC Bioinformatics 12, no. 1: 1–16. 10.1186/1471-2105-12-231. 21663684 PMC3212839

[eva70293-bib-0025] Korneliussen, T. S. , A. Albrechtsen , and R. Nielsen . 2014. “ANGSD: Analysis of Next Generation Sequencing Data.” BMC Bioinformatics 15, no. 1: 1–13. 10.1186/s12859-014-0356-4. 25420514 PMC4248462

[eva70293-bib-0026] Lamperth, J. S. , T. P. Quinn , and M. S. Zimmerman . 2017. “Levels of Stored Energy but Not Marine Foraging Patterns Differentiate Seasonal Ecotypes of Wild and Hatchery Steelhead ( *Oncorhynchus mykiss* ) Returning to the Kalama River, Washington.” Canadian Journal of Fisheries and Aquatic Sciences 74, no. 2: 157–167. 10.1139/cjfas-2016-0018.

[eva70293-bib-0065] Lewis, S. K. 2013. Reconnecting Aquatic Habitats: Validating Historical Habitat Use by Anadromous Fishes Using Telemetry and Stable Isotope Analysis Above Barriers. Master's Thesis, 118. Oregon State University.

[eva70293-bib-0027] Li, H. 2011a. “A Statistical Framework for SNP Calling, Mutation Discovery, Association Mapping and Population Genetical Parameter Estimation From Sequencing Data.” Bioinformatics 27, no. 21: 2987–2993. 10.1093/bioinformatics/btr509. 21903627 PMC3198575

[eva70293-bib-0028] Li, H. 2011b. “Improving SNP Discovery by Base Alignment Quality.” Bioinformatics 27, no. 8: 1157–1158. 10.1093/bioinformatics/btr076. 21320865 PMC3072548

[eva70293-bib-0029] Li, H. 2013. “Aligning Sequence Reads, Clone Sequences and Assembly Contigs With BWA‐MEM.” arXiv. 10.48550/arXiv.1303.3997.

[eva70293-bib-0030] Loomis, D. , J. Raasch , R. Thompson , and B. Ryan . 2003. A Radio Telemetry Study of Steelhead in the North Umpqua River Basin 1998–2001. Umpqua Watershed District: Oregon Department of Fish and Wildlife. https://nrimp.dfw.state.or.us/DataClearinghouse/default.aspx?pn=ViewFile&att=ODFW%2FODFW_41483_2_Radio+Telemetry+of+Steelhead+in+the+North+Umpqua+River2003.pdf.

[eva70293-bib-0031] McGie, A. M. 1994. Stock‐Recruitment in Summer‐Run Steelhead of the North Umpqua River, Oregon Information Reports 94–5. Oregon Department of Fish and Wildlife. https://digitalcollections.library.oregon.gov/nodes/view/125861.

[eva70293-bib-0032] Miller, M. R. , J. P. Brunelli , P. A. Wheeler , et al. 2012. “A Conserved Haplotype Controls Parallel Adaptation in Geographically Distant Salmonid Populations.” Molecular Ecology 21, no. 2: 237–249. 21988725 10.1111/j.1365-294X.2011.05305.x PMC3664428

[eva70293-bib-0033] Miller, M. R. , J. P. Dunham , A. Amores , W. A. Cresko , and E. A. Johnson . 2007. “Rapid and Cost‐Effective Polymorphism Identification and Genotyping Using Restriction Site Associated DNA (RAD) Markers.” Genome Research 17, no. 2: 240–248. 10.1101/gr.5681207. 17189378 PMC1781356

[eva70293-bib-0034] Mimura, M. , T. Yahara , D. P. Faith , et al. 2017. “Understanding and Monitoring the Consequences of Human Impacts on Intraspecific Variation.” Evolutionary Applications 10, no. 2: 121–139. 10.1111/eva.12436. 28127389 PMC5253428

[eva70293-bib-0035] Moore, J. W. , M. McClure , L. A. Rogers , and D. E. Schindler . 2010. “Synchronization and Portfolio Performance of Threatened Salmon.” Conservation Letters 3, no. 5: 340–348. 10.1111/j.1755-263X.2010.00119.x.

[eva70293-bib-0036] Moyle, P. B. , J. A. Israel , and S. E. Purdy . 2008. Salmon, Steelhead, and Trout in California. University of California, Davis: Center for Watershed Sciences. https://docs.streamnetlibrary.org/CoastalCutthroatData/sn600356.pdf.

[eva70293-bib-0037] National Interagency Fire Center (NIFC) . 2025. Wildland Fire Management Research, Development, and Application, (WFM RD&A) Interagency Fire Perimeter History. https://data‐nifc.opendata.arcgis.com/datasets/nifc::interagencyfireperimeterhistory‐all‐years‐view/about.

[eva70293-bib-0038] ODFW . 2022. 2022 Assessment of Naturally Produced Summer Steelhead in the Umpqua River Basin, Science Bulletin 2022–1. Oregon Department of Fish and Wildline. https://www.dfw.state.or.us/fish/crp/docs/north_umpqua_summer_steelhead/2022_NU_StS_Assessment_FINAL.pdf.

[eva70293-bib-0039] ODFW . 2023. All Angling on North Umpqua River and Tributaries Closed July 31–Nov. 30 Accessed October 17, 2025. https://www.dfw.state.or.us/news/2023/07_Jul/072823.asp.

[eva70293-bib-0040] ODFW . 2025a. North Umpqua Hydroelectric Project (FERC No. 1927) Settlement Agreement Section 19.2 Long‐Term Monitoring and Predator Control Study Annual Report for 2024. Oregon Deparment of Fish and Wildlife.

[eva70293-bib-0041] ODFW . 2025b. Summer Steelhead at Winchester Dam. https://www.dfw.state.or.us/fish/fish_counts/winchester/historical/summer%20steelhead.pdf.

[eva70293-bib-0042] ODFW . 2025c. Winter Steelhead at Winchester Dam. https://www.dfw.state.or.us/fish/fish_counts/winchester/historical/winter%20steelhead.pdf.

[eva70293-bib-0043] Palmer, T. 2014. Field Guide to Oregon Rivers. Oregon State University Press.

[eva70293-bib-0044] Pearse, D. E. , N. J. Barson , T. Nome , et al. 2019. “Sex‐Dependent Dominance Maintains Migration Supergene in Rainbow Trout.” Nature Ecology & Evolution 3, no. 12: 1731–1742. 10.1038/s41559-019-1044-6. 31768021

[eva70293-bib-0045] Prince, D. J. , S. M. O'Rourke , T. Q. Thompson , et al. 2017. “The Evolutionary Basis of Premature Migration in Pacific Salmon Highlights the Utility of Genomics for Informing Conservation.” Science Advances 3, no. 8: e1603198. 10.1126/sciadv.1603198. 28835916 PMC5559211

[eva70293-bib-0046] Quinn, T. P. 2007. “The Behavior and Ecology of Pacific Salmon and Trout.” In The Behavior and Ecology of Pacific Salmon and Trout. University of British Columbia Press. 10.59962/9780774854610.

[eva70293-bib-0047] Quinn, T. P. , P. McGinnity , and T. E. Reed . 2016. “The Paradox of ‘Premature Migration’ by Adult Anadromous Salmonid Fishes: Patterns and Hypotheses.” Canadian Journal of Fisheries and Aquatic Sciences 73, no. 7: 1015–1030.

[eva70293-bib-0048] Quinn, T. P. , T. R. Seamons , L. A. Vøllestad , and E. Duffy . 2011. “Effects of Growth and Reproductive History on the Egg Size–Fecundity Trade‐Off in Steelhead.” Transactions of the American Fisheries Society 140, no. 1: 45–51. 10.1080/00028487.2010.550244.

[eva70293-bib-0049] Quinones, R. M. , T. E. Grantham , B. N. Harvey , et al. 2015. “Dam Removal and Anadromous Salmonid (Oncorhynchus spp.) Conservation in California.” Reviews in Fish Biology and Fisheries 25, no. 1: 195–215.

[eva70293-bib-0050] R Core Team . 2024. R: A Language and Environment for Statistical Computing. Foundation for Statistical Computing. https://www.R‐project.org/.

[eva70293-bib-0051] Reeves, G. H. , F. H. Everest , and J. D. Hall . 1987. “Interactions Between the Redside Shiner ( *Richardsonius balteatus* ) and the Steelhead Trout (Salmo Gairdneri) in Western Oregon: The Influence of Water Temperature.” Canadian Journal of Fisheries and Aquatic Sciences 44, no. 9: 1603–1613. 10.1139/f87-194.

[eva70293-bib-0052] Sandercock, F. K. 1991. Pacific Salmon Life Histories. UBC Press.

[eva70293-bib-0053] Schindler, D. , R. Hilborn , B. Chasco , et al. 2010. “Population Diversity and the Portfolio Effect in an Exploited Species.” Nature 465, no. 3: 609–612. 10.1038/nature09060. 20520713

[eva70293-bib-0054] Schleinitz, D. , J. K. DiStefano , and P. Kovacs . 2011. “Targeted SNP Genotyping Using the TaqMan Assay.” In Disease Gene Identification: Methods and Protocols, edited by J. K. DiStefano , 77–87. Humana Press. 10.1007/978-1-61737-954-3_6. 21204028

[eva70293-bib-0055] Spencer, L. 2017. A Temporary Refuge. Patagonia.

[eva70293-bib-0056] Thompson, T. Q. , M. R. Bellinger , S. M. O’Rourke , et al. 2019. “Anthropogenic Habitat Alteration Leads to Rapid Loss of Adaptive Variation and Restoration Potential in Wild Salmon Populations.” Proceedings of the National Academy of Sciences 116, no. 1: 177–186. 10.1073/pnas.1811559115. PMC6320526 30514813

[eva70293-bib-0057] Twardek, W. M. , T. O. Gagne , L. K. Elmer , S. J. Cooke , M. C. Beere , and A. J. Danylchuk . 2018. “Consequences of Catch‐And‐Release Angling on the Physiology, Behaviour and Survival of Wild Steelhead *Oncorhynchus mykiss* in the Bulkley River, British Columbia.” Fisheries Research 206: 235–246. 10.1016/j.fishres.2018.05.019.

[eva70293-bib-0062] U.S. Geological Survey . 2025. “National Water Information System data available on the World Wide Web (USGS Water Data for the Nation).” http://waterdata.usgs.gov/nwis/.

[eva70293-bib-0058] Waples, R. S. , M. J. Ford , K. Nichols , et al. 2022. “Implications of Large‐Effect Loci for Conservation: A Review and Case Study With Pacific Salmon.” Journal of Heredity 113, no. 2: 121–144. 10.1093/jhered/esab069. 35575083

[eva70293-bib-0059] Weigel, D. E. , P. J. Connolly , K. D. Martens , and M. S. Powell . 2013. “Colonization of Steelhead in a Natal Stream After Barrier Removal.” Transactions of the American Fisheries Society 142, no. 4: 920–930. 10.1080/00028487.2013.788560.

[eva70293-bib-0060] Withler, I. L. 1966. “Variability in Life History Characteristics of Steelhead Trout (Salmo Gairdneri) Along the Pacific Coast of North America.” Journal of the Fisheries Research Board of Canada 23, no. 3: 365–393. 10.1139/f66-031.

[eva70293-bib-0061] Wright, M. J. , M. Hurson , K. A. Robinson , D. A. Patterson , and J. G. Venditti . 2024. “A Typology of Potential Hydraulic Barriers to Adult Salmon Migration in a Bedrock River.” Canadian Journal of Fisheries and Aquatic Sciences 82: 1–19.

