# Supplementary material for: Genetic Analysis Reveals Climate Exposure and Conservation Opportunities for a Vulnerable Life History in Steelhead ( Oncorhynchus mykiss )
Source: Evol Appl. 2026 Jul 10;19(7):e70293. doi: 10.1111/eva.70293 (PMC13354926; doi:10.1111/eva.70293)

**Supplemental Material for “Genetic analysis reveals climate exposure and conservation opportunities for a vulnerable life history in steelhead (Oncorhynchus mykiss)”**

**Table S1.** Species validation sample information. Data from the samples listed in the table were downloaded from NCBI and used as known-species samples in the principal component analysis used to identify off-target juvenile samples collected in the North Umpqua.

| **Species** | **NCBI Acces** **sion** | **Publication** | **Publication DOI** |
| --- | --- | --- | --- |
| Cutthroat | SRR8690513 | Bay et al. (2019) | DOI:10.1111/mec.15097 |
| Cutthroat | SRR8690442 | Bay et al. (2019) | DOI:10.1111/mec.15097 |
| Chinook | SRR5372164 (Read 1) SRR5371881 (Read 2) | Prince et al. (2017) | [DOI: 10.1126/sciadv.16031](https://doi.org/10.1126/sciadv.1603198) |
| Chinook | SRR5372145 (Read 1) SRR5371862 (Read 2) | Prince et al. (2017) | [DOI: 10.1126/sciadv.16031](https://doi.org/10.1126/sciadv.1603198) |
| Coho | SRR6180134 | Cambell et al. (2017) | DOI:10.1002/ece3.3492 |
| Coho | SRR6180139 | Cambell et al. (2017) | DOI:10.1002/ece3.3492 |
| Steelhead | SRR5373568 (Read 1) SRR5373684 (Read 2) | Prince et al. (2017) | [DOI: 10.1126/sciadv.16031](https://doi.org/10.1126/sciadv.1603198) |
| Steelhead | SRR5373529 (Read 1) SRR5373645 (Read 2) | Prince et al. (2017) | [DOI: 10.1126/sciadv.16031](https://doi.org/10.1126/sciadv.1603198) |

| **Table S2.** Primer sequence information for the qPCR assay for steelhead run timing genotype. | |
| --- | --- |
| **Primer name** | **Primer sequence** |
| Fwd Primer | GCAGGACTTGGATAACACAGAATGT |
| Rev Primer | TCGTGGTTATATCTACAGTACAGTTCGT |
| FAM Probe (Winter-run allele) | CAATGCAAG**G**CTTAAA |
| VIC Probe (Summer-run allele) | CACAATGCAAG**A**CTTAAA |
| Context Sequence | TTGTTTTATTAAAACACAATGCAAG**[A/G]**CTTAAAACGAACTGTACTGTAGATA |

**Figure S1.** All juvenile samples collected from the North Umpqua in 2017, 2018, and 2019, shown by collection location. Off-target samples have not been removed; A) all years combined; B) samples divided by collection year.


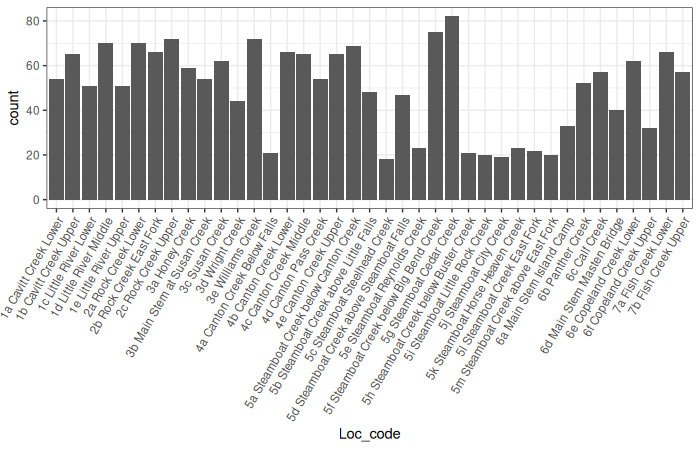


**A**

**B**
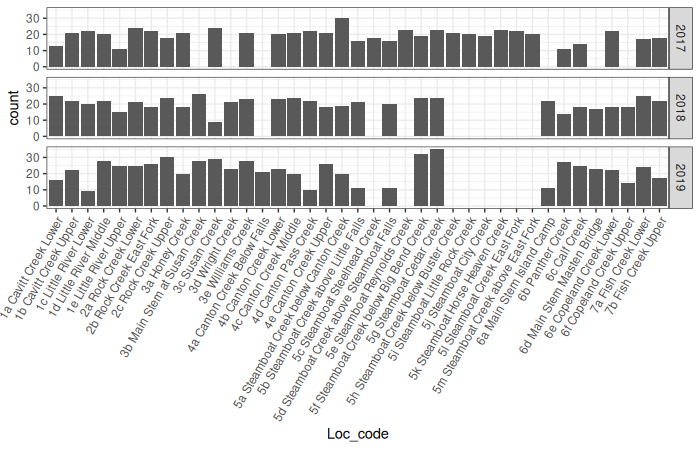


**Figure S2.** Species confirmation analyses. Principal component analysis of all juvenile salmonids collected in the North Umpqua as part of this study combined with data from known species listed in Supplemental Table 1. Samples were considered to be *O. mykiss* if at a negative position on both PC1 and PC2 (the tight lower left cluster). Cutthroat were defined as samples positioned in the positive numbers along PC2 (this likely includes some steelhead/cutthroat hybrids). Chinook and Coho salmon are the clusters to the right of 0.050 on PC1 and below 0.00 on PC2.


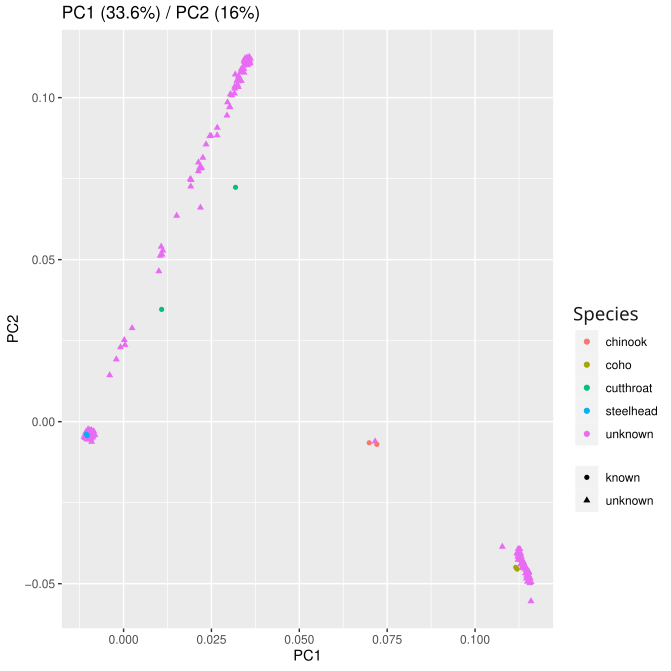


**Figure S3.** Proportion of off-target species at each location in 2017 and 2018; A) includes all off-target species; B) off-target species other than cutthroat have been excluded. Dashed line indicates 15% cutthroat cutoff for location inclusion in 2019 dataset.


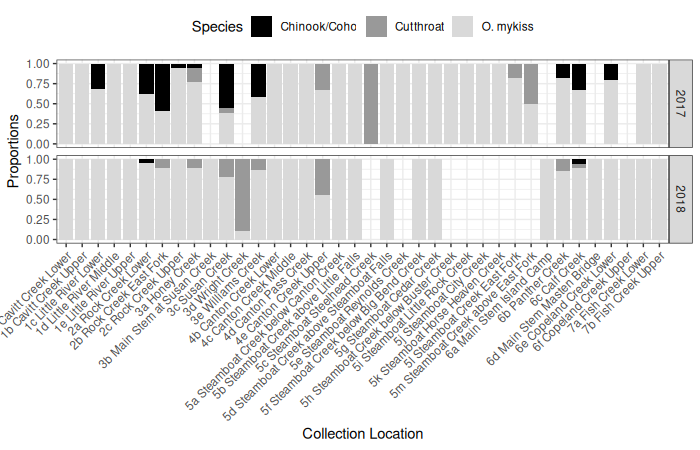


**A**


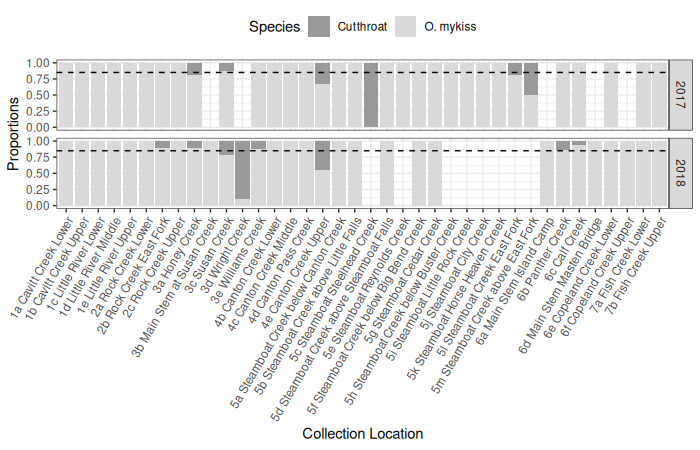


**B**

**Figure S4.** Stacked bar graph counts of run timing genotypes at each location and year.


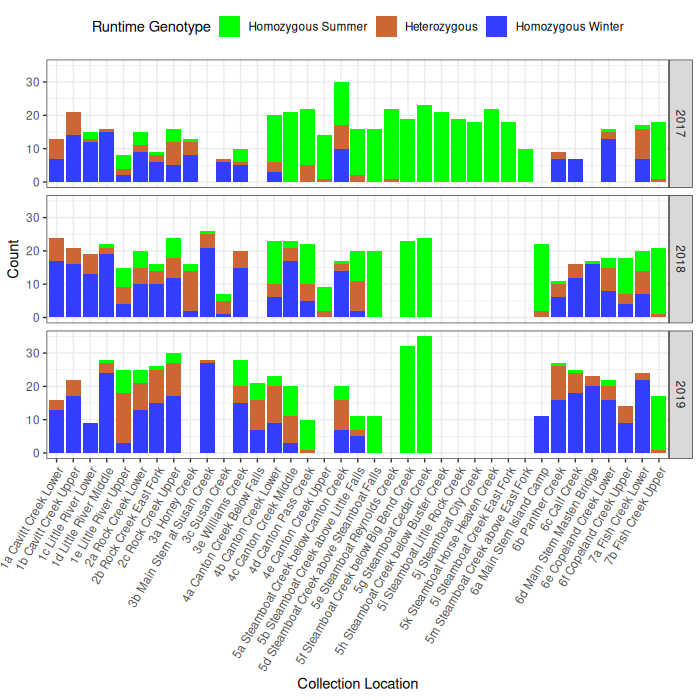


**Figure S5.** Principal Component Analysis of North Umpqua juveniles. All locations included except Fish Creek sites. Blue-colored outliers along PC2 are samples from the (6f) Copeland Creek Upper site.


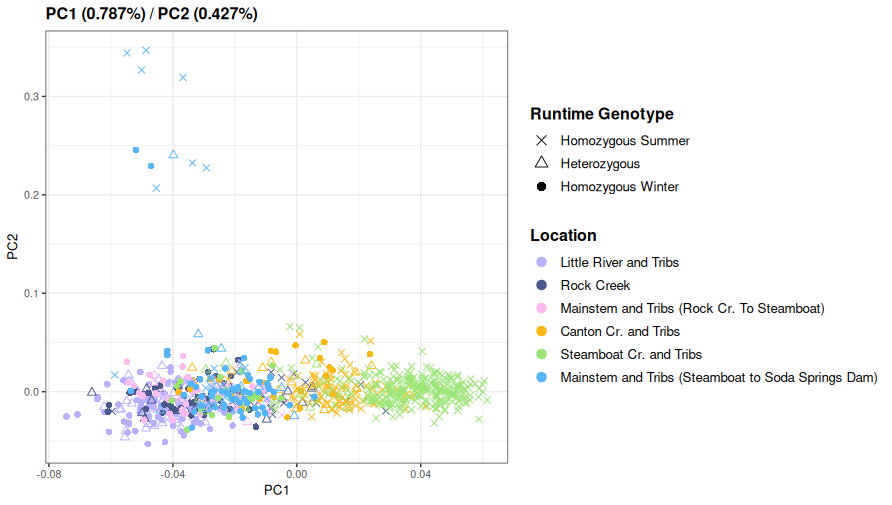


Figure S6. Water to air temperature regression analyses.


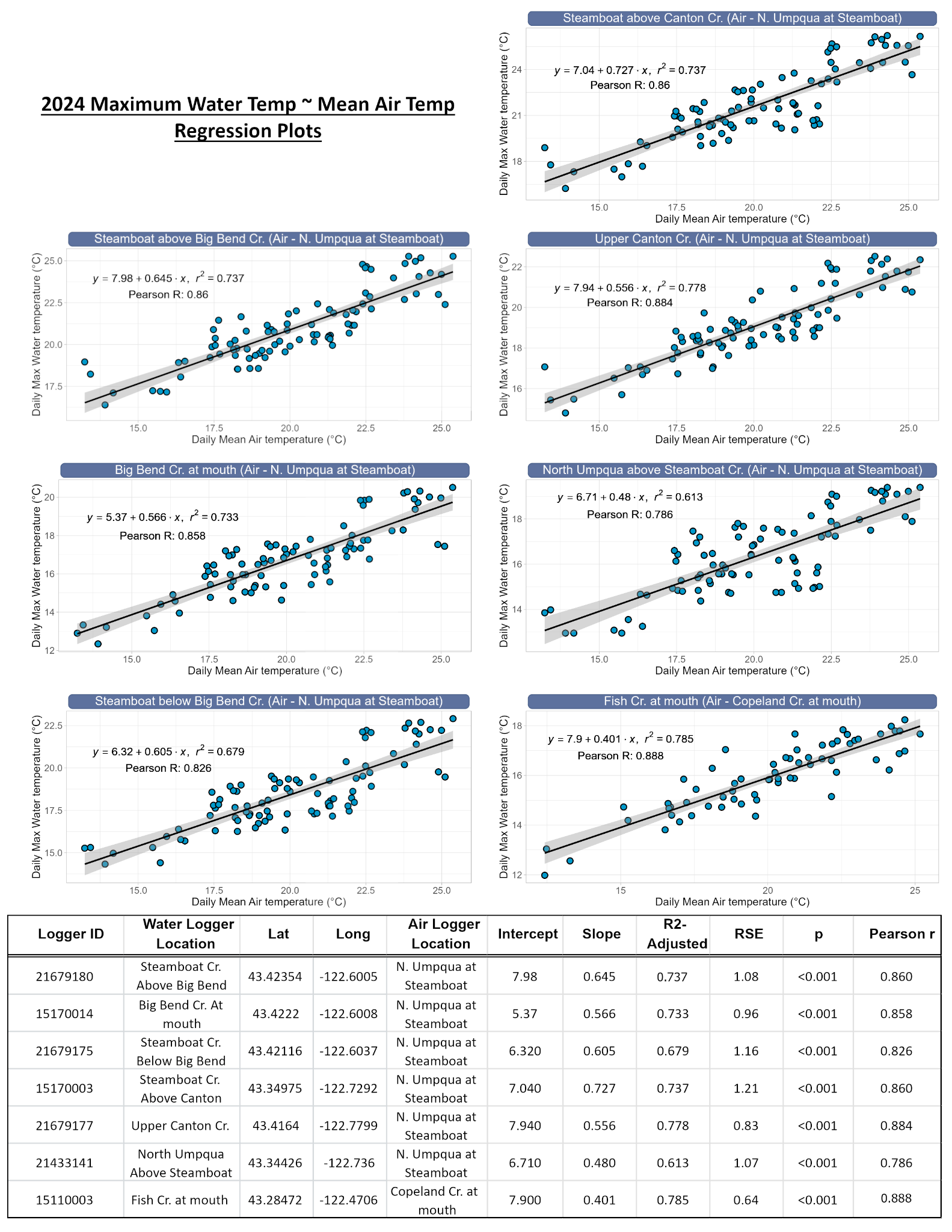

Supplement: Supplementary file 1 — Table S1: Species validation sample information. Data from the samples listed in the table were downloaded from NCBI and used as known‐species samples in the principal component analysis used to identify off‐target juvenile samples collected in the North Umpqua. Table S2: Primer sequence information for the qPCR assay for steelhead run timing genotype. Figure S1: All juvenile samples collected from the North Umpqua in 2017, 2018, and 2019, shown by collection location. Off‐target samples have not been removed; (A) all years combined; (B) samples divided by collection year. Figure S2: Species confirmation analyzes. Principal component analysis of all juvenile salmonids collected in the North Umpqua as part of this study combined with data from known species listed in Table S1. Samples were considered to be O. mykiss if at a negative position on both PC1 and PC2 (the tight lower left cluster). Cutthroat were defined as samples positioned in the positive numbers along PC2 (this likely includes some steelhead/cutthroat hybrids). Chinook and Coho salmon are the clusters to the right of 0.050 on PC1 and below 0.00 on PC2. Figure S3: Proportion of off‐target species at each location in 2017 and 2018; (A) includes all off‐target species; (B) off‐target species other than cutthroat have been excluded. Dashed line indicates 15% cutthroat cutoff for location inclusion in 2019 dataset. Figure S4: Stacked bar graph counts of run timing genotypes at each location and year. Figure S5: Principal Component Analysis of North Umpqua juveniles. All locations included except Fish Creek sites. Blue‐colored outliers along PC2 are samples from the (6f) Copeland Creek Upper site. Figure S6: Water to air temperature regression analyzes. [file EVA-19-e70293-s001.docx]
